# Discovery of anti-Formin-like 1 protein (FMNL1) antibodies in membranous nephropathy and other glomerular diseases

**DOI:** 10.1038/s41598-022-17696-w

**Published:** 2022-08-11

**Authors:** Maurizio Bruschi, Andrea Cavalli, Solange Moll, Giovanni Candiano, Leonardo Scapozza, Jigar J. Patel, John C. Tan, Ken C. Lo, Andrea Angeletti, Gian Marco Ghiggeri, Marco Prunotto

**Affiliations:** 1 Laboratory of Molecular Nephrology, IRCCS Istituto Giannina Gaslini, Via G. Gaslini, 5, 16147 Genoa, Italy; 2 Institute for Research in Biomedicine, Università Della Svizzera Italiana, Bellinzona, Switzerland; 3 Swiss Institute of Bioinformatics, Lausanne, Switzerland; 4 Department of Pathology, University Hospital of Geneva, Geneva, Switzerland; 5 Nimble Therapeutics, Madison, WI USA; 6 Division of Nephrology, Dialysis and Transplantation, IRCCS Istituto Giannina Gaslini, Genoa, Italy; 7 Institute of Pharmaceutical Sciences of Western Switzerland, University of Geneva, Geneva, Switzerland

**Keywords:** Peptides, Bioinformatics, Autoimmunity, Membranous nephropathy, Biomarkers

## Abstract

Evidence has shown that podocyte-directed autoantibodies can cause membranous nephropathy (MN). In the present work we investigated sera of MN patients using a high-density peptide array covering the whole coding sequences of the human genome encompassing 7,499,126 tiled peptides. A panel of 21 proteins reactive to MN sera were identified. We focused our attention on Formin-like 1 (FMNL1), a protein expressed by macrophages in MN patients tissues. High levels of anti-FMNL1 IgG4 were demonstrated in sera of MN patients with an orthogonal methodology (ELISA) contemporary demonstrating FMNL1 positive cells in kidney co-staining with CD68 in glomeruli. High levels of circulating anti-FMNL1 IgG4 were associated with lack of remission of proteinuria, potentially indicating that autoantibodies directed against cells other than podocytes, involved in tissue repair, might play a role in MN disease progression. High serum levels of anti-FMNL1 IgGs were also observed in other non-autoimmune glomerolonephrites, i.e. idiopathic and genetic FSGS, IgAGN. These findings are suggestive of a broader role of those autoantibodies in other glomerular disease conditions.

## Introduction

The identification of the root causes of diseases is one of the main goal of medicine. In the context of autoimmune disorders, this quest relies on the recognition of the pathogenetic role of circulating autoantibodies directed against disease-specific autoantigens. Membranous nephropathy (MN) is a condition associated with formation of nephrotoxic autoantibodies targeting the glomerular basement membrane. The recent identification of circulating pathogenic autoantibodies^1–3^ and their related antigens^4–6^ in MN has represented a real breakthrough in autoimmune disease mechanism understanding. These autoantibodies were discovered in serum or eluted from laser-microdissected glomeruli of affected patients and characterized by performing western blot and mass spectrometry.

Two major autoantibodies directed against antigens expressed by podocytes, i.e*.* anti-phospholipase A2 receptor (PLA2R1) and anti-Thrombospondin type 1 domain-containing 7A (THSD7A), were identified in 2009 and in 2014 respectively, altogether accounting for approximately 60–70% of cases. More recently, novel additional antigens, i.e*.* Neural epidermal growth factor-like (NELL), semaphorin3B (SEMA3B), protocadherin 7 (PCDH7) and serine protease HTRA1^7^ have been described in glomeruli of the 15–20% of cases with PLA2R1 negative primary MN^4–6,8^, potentially completing the panel of autoantibodies causative of the disease^9^. Exostosin-1 and -2 (Ext 1–2)^4^ antigens have been identified within the glomerular immune deposits in renal biopsies of patients with secondary MN due to systemic lupus erythematosus (SLE).

Although the discovery of autoantibodies linked with MN explains per se the mechanism of disease onset, a clinical debate still exists in relation to their role in disease progression modulation. Indeed, 30–40% of patients with MN evolve to end-stage renal disease (ESRD) in within 5–15 years^10–12^. In a cohort of 285 MN patients our team discovered and recently documented the existence of circulating autoantibodies targeting anti-oxidant enzymes such as superoxide dismutase 2 (SOD2)^13,14^ that takes part of the protective response to an oxidative process^15,16^. Experimental models support this notion^15,16^.

In the present study, we investigated MN patient sera using a high-density peptide array encompassing 7,499,126 tiled peptides covering the whole protein sequences coded by the human genome. This technology has been extensively used to characterize binding epitopes of monoclonal antibodies^17^, for quantitative evaluation of protein kinase activity^18,19^ and to define autoantibody signatures in several diseases^20–22^. The designed array, coupled with a novel workflow for data analysis, was used to investigate the occurrence of autoantibody target peptide epitopes in baseline sera of highly characterised MN patients.

Subsequent to a short proof of concept study using PLA2R1 epitopes, we set to extend the analysis to the whole human proteome. Those findings were subsequently correlated to clinical outcome. Among all those novel target antigens, we focused on Formin Protein Formin-like 1 (FMNL1), the protein having the strongest signal intensity with the highest − Log10 *P*-values in PLA2R1 negative MN patients initially hypothesized being expressed by podocytes. Validation of peptide array findings was carried out by adopting an orthogonal methodology (ELISA) using FMNL1 recombinant protein. Immunohistochemistry on renal biopsies showed FMNL1 expression being limited to macrophages. In addition to MN sera, other categories of primary glomerulonephritides (GNs) were analysed for circulating anti-FMNL1 antibodies.

## Results

### Study design and patient description

This study was designed to assess novel circulating autoantibodies in MN and their potential correlation with the clinical outcome.

**Scheme 1:**
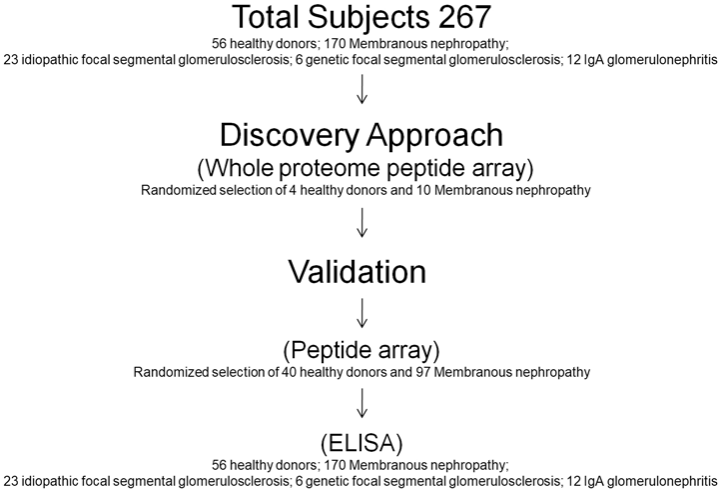
Scheme of the numbers of subjects used in the different phases of the study. The discovery phase of novel autoantibodies was performed in a subset of 4 healthy donors and 10 MN patients randomly extracted from the whole sample dataset. Then, the validation of results deriving from the previous phase was carried out first using the validation peptide array in 40 healthy donors and 97 MN patients, and finally, the selected autoantibodies were validated using ELISA in all subjects of the study.

The study included a total of 56 healthy donors and 211 patients (170 with MN, 23 with idiopathic focal segmental glomerulosclerosis (FSGS), 6 with genetic FSGS, 12 with IgA glomerulonephritis). Samples were utilized at different phases of the study as it follows (see Scheme 1): (a) the fishing of new antibodies with the peptide array (discovery approach) was initially performed in a subset of 4 healthy and 10 MN patients randomly extracted from the whole sample dataset; (b) validation of results deriving from step 1 was carried out in 40 healthy donors and 97 MN patients; (c) all healthy donors and different pathology subsets (n 267) were utilized for comparison of results obtained with quantitative tests (ELISA) on selected antibodies. Clinical characteristics of healthy, MN, and other categories of patients with glomerulonephritis are reported in Tables 1 and 2.

**Table 1:** Clinical characteristics of healthy donor and idiopathic membranous nephropathy (MN) patients utilized in the discovery and validation study.

	Anti-PLA2R1 positive	Anti-PLA2R1 negative	Healthy
Subjects	90	80	56
Sex (M/F)	60/30	50/30	30/26
Age (year)	58 (47–67)	51 (42–67)	45 (31–51)
Anti-PLA2R1 (RU/ml)	139.9 (62.1)	3 (1.2–5.4)	–
Proteinuria at T_0_ (g/d)	6.5 (4.7–9.8)	5.5 (2.8–8.0)	0.1 (0.08–0.12)
Proteinuria at T_12_ (g/d)	1.3 (0.5–4.1)	1.3 (0.3–3.3)	
EPI-KDK at T_0_ (ml/min/1.73m^2^)	81.9 (47.6–100.0)	88.6 (68.5–104.0)	100.0 (90.1–118.4)
EPI-KDK at T_12_ (ml/min/1.73m^2^)	87.7 (45.5–101.8)	95.8 (56.2–105.5)	
Therapy (No Immuno/Cytotoxic/Rituximab/Other)	21/45/5/19	30/32/9/9	
Outcome at T_12_ (Complete Remission/Partial Remission /Persistence of Proteinuria)*	14/58/18	21/40/19	

Continuous data were reported as median and interquartile range.

*Complete Remission = proteinuria < 0.3 g/d; Partial Remission = proteinuria between 0.3–3.5 g/d; Persistence of proteinuria = proteinuria > 3.5 g/d. The data were reported as median and interquartile range.

**Table 2:** Clinical characteristics of different patient subsets utilizedin this study.

	Genetic FSGS	Idiopathic FSGS	IgAGN
Subjects	6	23	12
sex (M/F)	4/2	14/10	8/4
Age (years)	8 (5–12)	16 (8–26)	43 (36–58)
Proteinuria at T_0_ (g/d)	4.5 (2.6–6.8)	6.5 (4.8–8.0)	3.8 (2.2–4.6)
Proteinuria at T_12_ (g/d)	3.6 (3–8)	2.3 (1.3–3.3)	0.6 (0.3–1.2)
EPI-KDK at T_0_ (ml/min/1.73m^2^)	91.9 (67.6–110.0)	78.6 (58.5–94.0)	86 (56–112)
EPI-KDK at T_12_ (ml/min/1.73m^2^)	97.7 (65.5–102.8)	85.8 (66.2–100.5)	96 (63–102)
Theraphy (No Immuno/Cytotoxic/Rituximab/Other)	6/0/0/6	0/9/9/8	12/0/0/12

Continuous data were reported as median and interquartile range.

### Whole proteome peptide discovery approach

**Figure 1:**
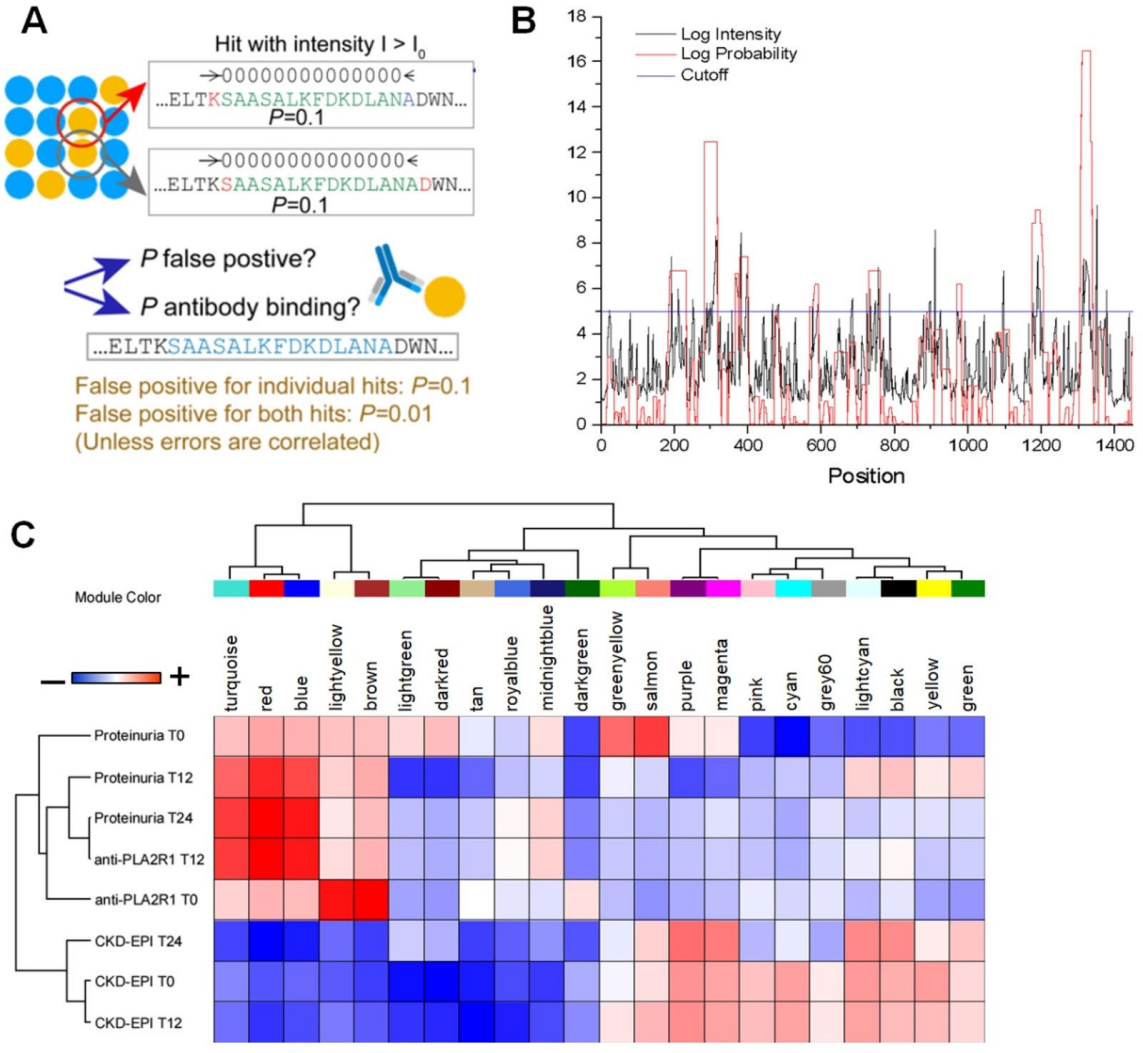
Method validation. (**A**) Illustration of the S-PIE underlying principle. Let P be the probability that a peptide with a fluorescence intensity larger than some threshold (I0) is a false positive. Then for two consecutive peptides the probability of both being false positive is equal to P^2. The same holds for longer stretches, which enable the identification of most probable epitopes. (**B**) Two-dimensional plot of peptides fluorescence intensity and their S-PIE analysis. (**C**) Heatmap showing the module-trait weighted relationships between the identified modules in *Whole proteome peptide discovery array* and the trait indicator of samples. The color scale on the top shows a module-trait relationship from − 1 (blue) to 1 (red), where blue represents a perfect negative correlation and red a perfect positive correlation.

The number of samples to be investigated in the discovery approach (10 MN sera, 4 healthy donors) was set out a priori considering the maximal capacity of the peptide array to trace the whole genome panel of 7.499.126 peptides (see “Material and Methods” section) made up of 16 overlapping amino acids each (Fig. 1A). In the discovery phase, two independent mathematical models were devised that allowed the identification of peptides with a significant probability to make a distinction between MN and healthy sera on the basis of the fluorescence intensity (S-PIE) and to correlate the intensity of reaction with the clinical outcome in MN patients (WGCNA). Specifications of each individual approach are reported in “Material and Methods” section. Those methods were then applied in the subsequent phases of the present work.

S-PIE identified 8243 peptides (0.1% of the 7.499.126 panel) with R-ratio higher than 10^6^ that is the limit of the noise signal (Fig. 1A,B). Subsequently, all peptides with a similar ratio in at least 1 control were removed, resulting in 3831 remaining peptides. As a last filter, all sequences with low Shannon information (< 3.17) were discarded, to keep only 756 peptides (0.01% of the 7.499.126 panel).

In the WGCNA approach, the whole dataset of 7.499.126 peptides filtered for fluorescence intensity (i.e*.*, 8.676 peptides with median intensity MN sera > healthy sera) were clustered into 22 modules characterized by peptides with a similar fluorescence signal intensity to which it was assigned an arbitrary colour (Fig. 1C). Among these, red, turquoise and blue modules showed the higher Spearman correlation with the clinical outcome at T_12_ and T_24_ (red^T12^ = 0.74; red^T24^ = 0.92; turquoise^T12^ = 0.53; turquoise^T24^ = 0.67; blue^T12^ = 0.63; blue^T24^ = 0.8) for a total of 277 peptides (PLA2R1 peptides excluded). Overall, the discovery phase (S-PIE and WGCNA) generated a total of 1.000 peptides (756 S-PIE, 277 WGCNA, 33 common to both) that identified 467 proteins.

### Peptide array technology identifies prior known PLA2R1 epitopes

High-density peptide technology validation was carried out using PLA2R1, the major recognized autoantigen of MN^1^. S-PIE recognized 9 peptides within the constitutive 2765 peptides of PLA2R1 with R-ratio larger than the cut-off (> 10^6^) that included all the epitopes of the protein (CysR, FNII, CTDL1-8). WGCNA recognized 96 and 108 peptides of the first five CTLD domains of PLA2R1 that correlated with the clinical outcome (proteinuria at 12 or 24 months) (Supplemental Table 2); we also confirmed that CysR was not included in the epitope predictor panel^23^. Fisher's enrichment analysis of PLA2R1 epitopes in the 22 modules identified by WGCNA highlighted epitopes associated with proteinuria outcome (Supplemental Fig. 1). The overlapping peptides obtained using both analysis (S-PIE and WGCNA) were then modelled on a 3D cryo-EM model^24^ (courtesy of Dr. Paul Brenchley, Supplemental Fig. 2). Interestingly, all peptides were accessible, i.e. located on the protein surface, and in our study mapped respectively to the CTLD1, CTLD3 and CTLD6 domains: in position 289 and 307 (CTLD1); in position 1080 (CTLD6), as postulated by Cui et al. using genome wide association study^25^; in position 285 and 1130, or in the CTLD3 domain in position 590, as previously reported by Fresquet et al*.*; and finally in position 613 using chimeric PLA2R1 constructs^24^.

### Validation array identified novel autoantibodies in MN sera

**Figure 2:**
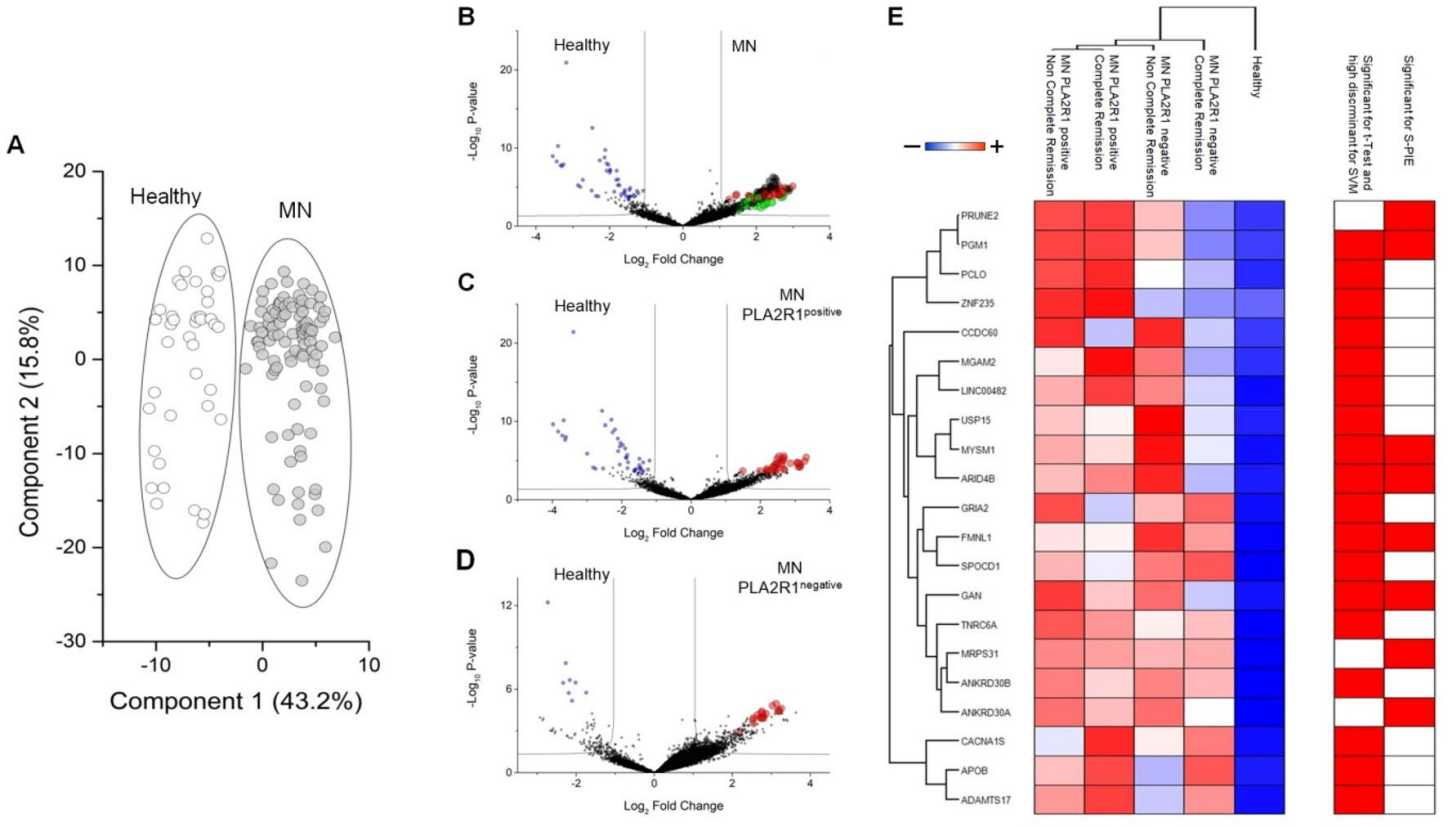
Validation Peptides array. (**A**) Scatter plot of Multidimensional Scaling (MDS) analysis of validation peptide intensity profiles of healthy (open circles) and MN (grey circles) patients. Symbols and ellipses indicate respectively each sample and the 95% confidence interval of the two conditions. Visual inspection of the scatter plot demonstrates the ability of their intensity value to clearly discriminate between the healthy and MN patient conditions. (**B–D**) Volcano plot of statistically significant peptides in the comparison between healthy and all MN patients (**B**) or healthy and MN anti-PLA2R1 positive (**C**) or healthy and MN anti-PLA2R1 negative (**D**). Small Black, blue, red, green and large black circles show respectively not statistically significant peptides, statistically up-regulated in healthy or in MN patients (positive or negative for anti-PLA2R1), selected from S-PIE, WGCNA and t-test analyses. The black line shows the cut-off of statistical significance. (**E**) Heatmap of 21 core proteins identified through the combined use of WGCNA, univariate statistical analysis, support vector machine learning and S-PIE analysis. In the heatmap, each row represents a protein and each column corresponds to a clinical condition. Normalized Z-scores of protein abundance are depicted by a pseudocolor scale with red indicating positive expression, white equal expression and blue negative expression compared to each protein value, whereas the dendrogram (on the top and left) displays the outcome of unsupervised hierarchical clustering analysis, placing similar sample/proteome profile values near each other. Visual inspection of the dendrogram and heat map demonstrates the ability of these proteins to clear discriminate between the different conditions.

To localize epitope binding sites for autoantibodies in the 467 proteins recognized in the initial phase, an independent peptide array was designed utilizing the 1000 identified peptides of 13 amino acids, modified at both edges for addition of 15 amino acids each, for an overall sequence of 43 amino acids. Eliminating redundant peptides, the new array consisted of 18.557 peptides of 16 amino acids each that covered the whole sequence of the 467 proteins identified above (Supplemental Table 1). Forty normal sera and 97 MN patients were tested. Fluorescence intensities were analysed by either S-PIE, multidimensional scaling (MDS), T-test analysis (Volcano plot), Support Vector Machine (SVM) and partial last squire discriminant analysis (PLS-DA) (Fig. 2). In particular, MDS analysis applied to fluorescence intensity of all peptides allowed the clear discrimination between healthy and MN patients (Fig. 2A). S-PIE analysis showed a total of 72 peptides with intensities higher than the cut-off, whereas the application of a T-test corrected for multiple interactions, PLS-DA and SVM identified 71, 33 and 19 peptides maximizing the discrimination between healthy and MN, healthy and anti-PLA2R1 positive and healthy andanti-PLA2R1 negative sera respectively (see Volcano plot in Fig. 2B–D).Their intensity profiles were visualised as heatmap (Supplemental Fig. 3). These 105 peptides identified 21 human proteins (Fig. 2E and Supplemental Table 3). The mean of the multiple intensity profiles for each protein is reported in Fig. 2E. All 21 proteins are expressed by human and/or mouse podocyte cells (Supplemental Fig. 4). In particular, the cellular origins of these proteins were 15% from plasma membranes, 34% from the cytosol, 24% from the nucleus, 12% were secreted, 3% from extracellular matrix and 12% from other organelles such as Golgi apparatus, endoplasmic reticulum or mitochondria^26,27^.

Among the 5 proteins out of the 21 described above identified by both methods, FMNL1 had a strong signal intensity with the highest -Log_10_ *P*-values in PLA2R1 negative MN patients (Fig. 2D,E, Supplemental Tables 1 and 3). Moreover, FMNL1 had the minor rank score and major VIP score in SVM and PLS-DA respectively in the discrimination between PLA2R1 negative MN patients and healthy donors. Based on this result, we decided to focus on FMNL1 protein. It is noteworthy that the FMNL1 peptides correspond to the region with the highest B-cell epitope score, according to the BepiPred-2.0 server (Supplemental Fig. 5). The region spanning amino acids 609 to 639 of FMNL1 belong to an unstructured loop region preceding FH2 domain that should be accessible to autoantibodies.

### Anti-FMNL1 IgG4 are present in sera of MN patients

**Figure 3:**
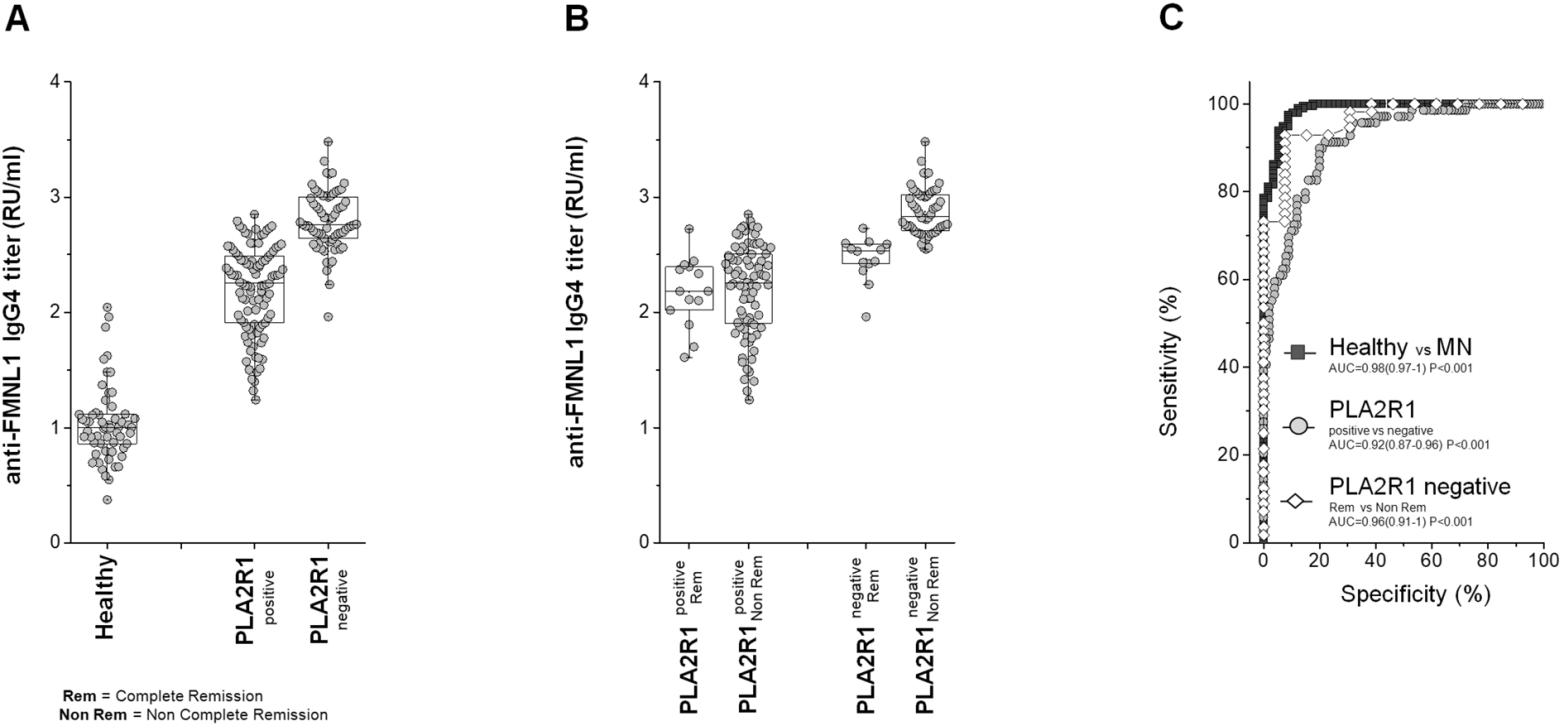
ELISA assay for anti-FMNL1 serum levels in patients with MN. (**A**) Box plot showing the median and interquartile range value of serum for anti-FMNL1 in all subjects of study. Anti-FMNL1 was statistically significant more abundant in MN patientsanti-PLA2R1 negative compared to healthy donors and MN anti-PLA2R1 positive (*P* < 0.0001). (**B**) Box plot of serum anti-FMNL1 in MN patients stratified in function of anti-PLA2R1 positivity and their range of proteinuria after one year from hospitalization (< 0.3 g/d complete remission; > 0.3 g/d no complete remission). Anti-FMNL1 was statistically significant more abundant in MN patients anti-PLA2R1 negative in no complete remission compared with all other MN patients (*P* < 0.0001). Moreover, no difference was present in anti-FMNL1 titer between MN patients anti-PLA2R1 positive in complete remission or not and anti-PLA2R1 negative in complete remission. (**C**) ROC curve analysis for FMNL1 assay.

Validation of the peptide array results was carried out using custom developed ELISA using FMNL1 recombinant protein. Prior to assessing the levels of anti-FMNL1 in sera of healthy donors or patients affected by MN, isotype characterisation was carried out. The vast majority of anti-FMNL1 antibodies present in patient with MN were, as expected, of IgG4 isotype (Supplemental Fig. 6). Levels of anti-FMNL1 IgG4 were then assessed in healthy and MN patient sera at diagnosis, showing a highly statistical difference (*P* < 0.0001). Anti-FMNL1 IgG4 were also more abundant in PLA2R1 negative MN patients compared to anti-PLA2R1 positive and in the former category, the antibody levels were higher in those patients who did not undergo remission during the follow up (T_12_)(respectively, anti-FMNL1 > 2.641 compared to anti-FMNL1 < 2.641 RU/ml [(OR) 10; (Cl) 3–35; *p* = 0.0002])(Fig. 3A,B). ROC analysis confirmed these differences (Fig. 3C).

### Anti-FMNL IgG tot are present in sera of other glomerulonephritis

**Figure 4:**
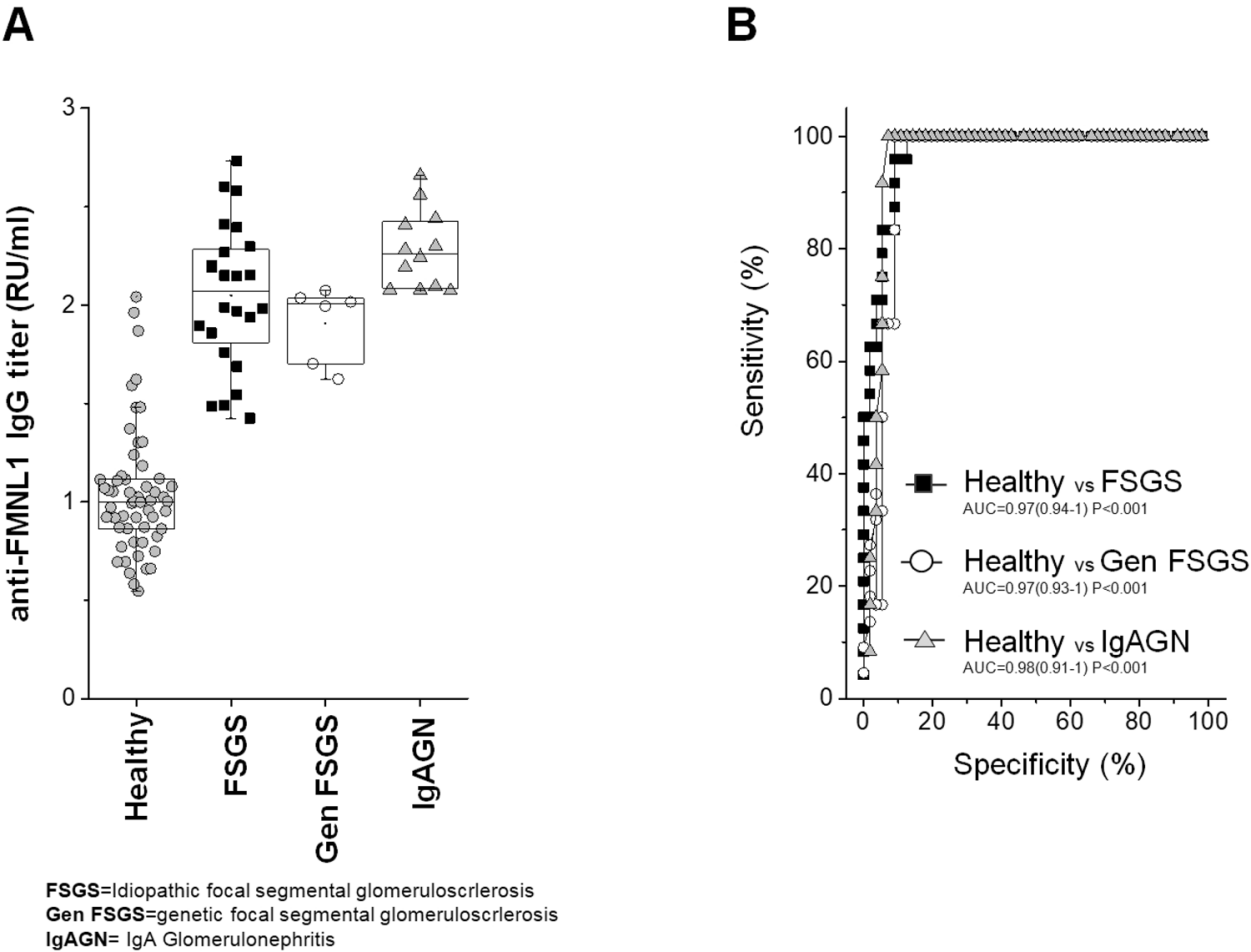
ELISA assay for anti-FMNL1serum levels in other glomerulonephritis. (**A**) The same ELISA modified for detection of total IgGs was utilized to test serum levels of anti-FMNL1 antibodies in patients affected by other hystologic and genetic forms of glomerulonephritis: 23 with idiopathic FSGS, 6 with genetic FSGS and12 with IgA glomerulonephritis. Anti-FMNL1 levels were statistically significant more abundant in all these groups compared with normal subjects (*P* < 0.0001). (**B**) ROC curve analysis for anti-FMNL1IgGs in comparison with normal controls.

To test anti-FMNL1 specificity for MN, small subgroups of patients affected by other hystologic and genetic forms of glomerulonephritis were tested (23 with idiopathic focal segmental glomerulosclerosis, 6 with genetic FSGS, 12 with IgA glomerulonephritis) (Table 2). In these cases, total IgG antibodies were determined Serum levels of anti-FMNL1 IgGs were in all cases higher than in normal control, particularly in patients with IgA GN (Fig. 4).

### MN sera recognize FMNL1 in cell lysates of macrophages

**Figure 5:**
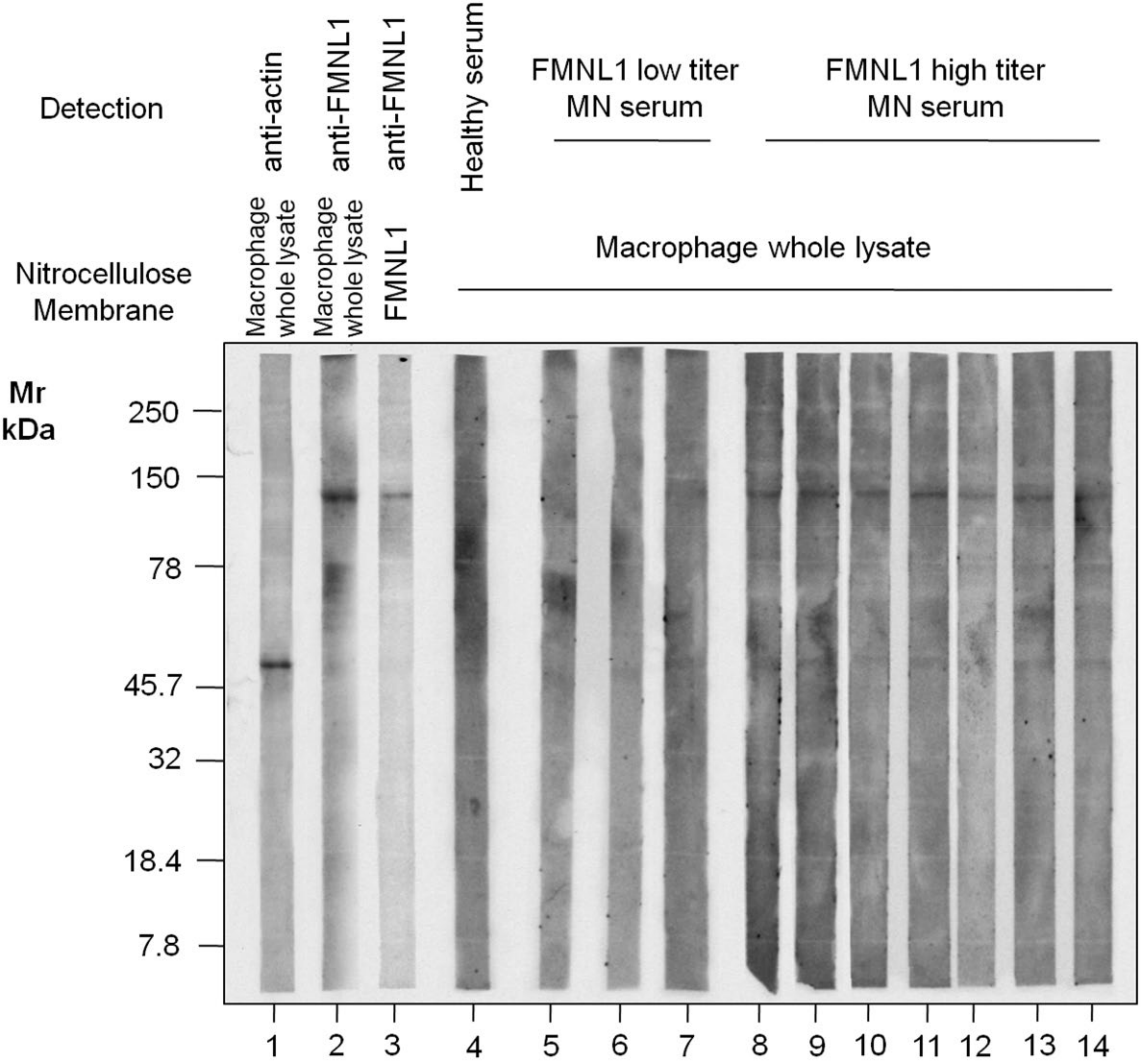
Characterization of macrophage antigens profile recognized by serum of healthy donors and MN patients. Representative western blot analysis of full length gel of macrophage antigen profile. Recombinant FMNL1 (lane 3) and macrophage whole lysate (lanes 1–2, and 4–12) were separated by SDS-PAGE in reducing conditions, transferred onto nitrocellulose membrane, and incubated with monoclonal anti-actin (lane 1), monoclonal anti-FMNL1 (lanes 2 and 3), a pool of healthy serum (lane 4) or MN patients low titer (lanes 5–7) or high titer (lanes 8–14) for anti-FMNL1 ELISA assay. Finally, membranes were developed, at the same time, with anti-Mouse IgG-HRP conjugated (lane 1–3) or anti-Human IgG4 HRP conjugated (4–14). IgG4 of MN patients with high titer for anti-FMNL1 in ELISA recognizes a band with the same molecular weight of recombinant FMNL1 protein. Besides, the same molecular weight band is recognized in macrophage whole lysate by monoclonal anti-FMNL1. In addition, IgG4 of MN patients recognizes a faint band with the same molecular weight of actin. Nitro cellulose membrane were cut perpendicular to the electrophoresis migration front to obtain a full length membrane strips of macrophage whole lysate and to allow the individually labeled and detection (at the same time) with anti-FMNL1, anti-actin and different healthy or patients sera.

Cell lysates of macrophages freshly isolated from whole blood of healthy subjects were blotted using sera from healthy subjects, FMNL1 negative or positive MN patients (Fig. 5). Western blot showed that FMNL1 positive MN patient sera recognized a band at 120 kDa, at the same molecular weight as recombinant FMNL1, thus confirming the specific presence of circulating autoantibodies against FMNL1, directed against macrophages in patient specific subset of MN patients. A lower band (above the 45,7 kDa marker) has been observed and might results from unspecific absorption of sera by actin, might represent a degradation product of FMNL1 or might potentially correspond to other novel antigens yet to be discovered present in human macrophage lysates.

### FMNL1 is expressed by macrophages within glomerular capillaries in glomerulonephritis

**Figure 6:**
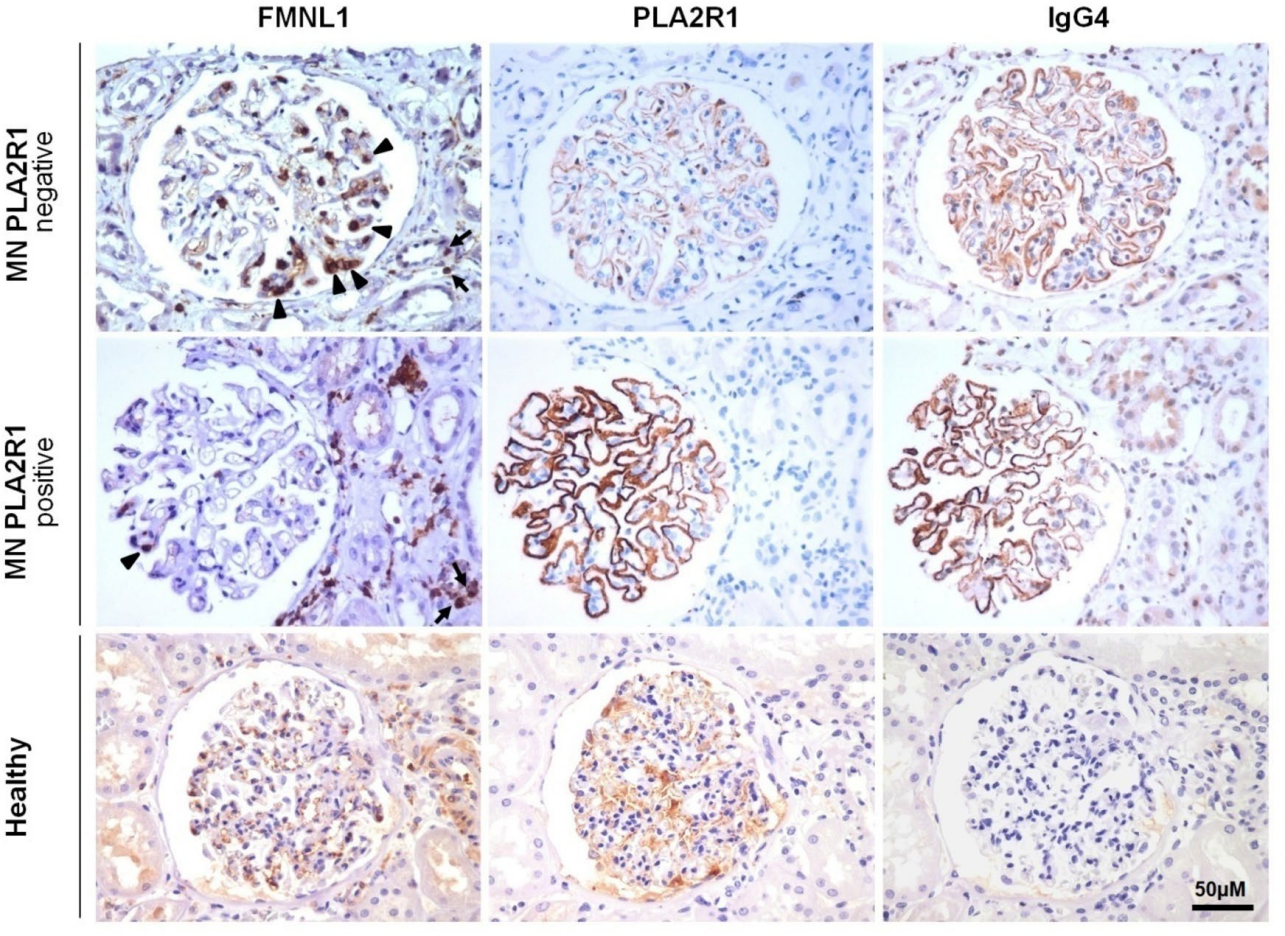
FMNL1 expression in MN biopsies and normal renal tissue. Immunohistochemistry was performed using FMNL1, PLA2R1 and IgG4 antibodies on PLA2R1 negative and positive MN biopsies and on normal renal tissue. In MN, FMNL1 was detected in circulating cells within glomerular capillaries (arrow heads) and in some cells in the interstitial space (arrows). Glomerular FMNL1 staining was distinctly different from staining of PLA2R1 (PLA2R1 positive MN) and IgG4 (PLA2R1 positive and negative MN) and was particularly expressed in PLA2R1 negative MN (patient with secondary MN due to lymphoma, patient 10) compared with PLA2R1 positive MN (patient 6). Arrowheads indicate FMNL1 positive glomerular circulating cells, arrows FMNL1 positive interstitial cells. Original magnification 320×, scale bar: 50 µm.

We assessed expression and localisation of FMNL1 positive cells in MN renal biopsies using immunohistochemistry. FMNL1 was expressed mainly in glomeruli (arrowheads) in circulating cells within the capillaries and was distinctly different from the membranous staining of PLA2R1 or IgG4. FMNL1 was also detected in some cells in the tubule-interstitial space (arrows, Fig. 6) of PLA2R1 negative MN biopsies. Only minimal FMNL1 staining could be observed in PLA2R1 positive MN biopsies. No FMNL1 positive cells could be detected in healthy tissue.

**Figure 7:**
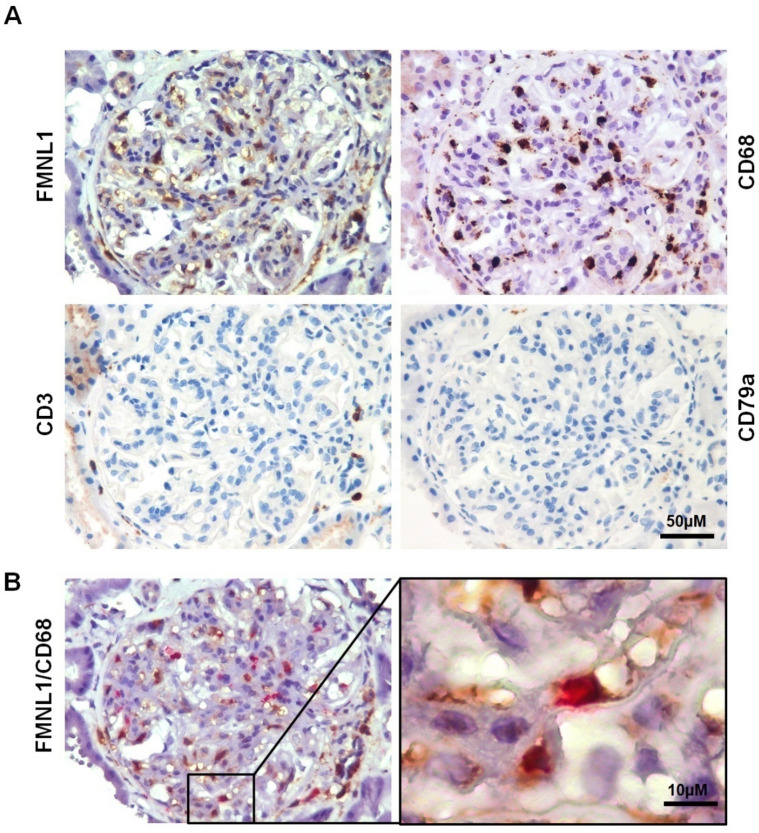
Characterization of FMNL1 expressing cells in renal biopsies. (**A**) Serial sections of PLA2R1 negative MN were immunostained with FMNL1, CD68 (monocyte/macrophage marker), CD3 (lymphocyte T marker) and CD79a (lymphocyte B marker) antibodies. Glomerular circulating cells expressing FMNL1 were CD68 positive and CD3 and CD79a negative. Original magnification 320x, scale bar: 50 µm. (**B)** PLA2R1 negative MN section was double immunostained with FMNL1 and CD68 Representative micrographs of glomerular circulating cells positively stained with FMNL1 (brown) and CD68 (red). Boxed area is enlarged in the right side of the figure. Magnification 320x, scale bar: 50 µm; Boxed area magnification 1600 x, scale bar:10 µm.

Immunohistochemistry using serial sections (Supplemental Table 4) showed cells expressing FMNL1 to be CD68 positive (monocytes/macrophages) and negative for CD3 and CD79a (T and B lymphocyte markers respectively) (Fig. 7A). Double immunostaining for FMNL1 and CD68 showed co-localization of these two proteins in monocytes/macrophages (Fig. 7B).

## Discussion

The identification of PLA2R1 as the main autoantigen of primary MN (65% of cases positive)^1^ and of other ancillary antigens, i.e*.* THSD7A^2^, NEP1^8^, NELL1^5^, SEMA3B^6^, PCDH7^9^ and HTRA1^7^ represented a significant advance in the definition of MN disease mechanism. It also allowed classifying disease subtypes enabling differential prognosis. All the above renal antigens/circulating autoantibodies have been identified by microelution from glomeruli and subsequent characterization by mass spectrometry.

In the present study, we implemented a different strategy for the discovery of novel antibodies, namely the incubation of MN patient sera using tiled peptide arrays, a technology extensively used in the pharmaceutical industry to characterize binding epitopes of monoclonal antibodies^17^ and for quantitative evaluation of protein kinase activity^18,19^. This technology using 7,499,126 tiled peptides encompassing the coding sequences of the whole human genome, has been profitably deployed for the identification of target epitopes in multiple sclerosis and narcolepsy^21^, non-small cell lung cancer^20^ and rheumatoid arthritis^22,28^. It is important to mention that, though incredibly powerful, tiled high-density peptide arrays have the inconvenience of being prone to a high false positive rate. A combined data analysis pipeline (S-PIE + WGCNA) was therefore devised to enable not only the identification of highly probable autoantibody target peptides, but also to correlate those highly probable epitopes to proteinuria at different time points. It is worth noting that the peptide technologies may add new discoveries to historical techniques based on western blot but it is not alternative. One example is the definition of PLA2R1 epitopes^24,25,29^ that was used to validate the whole workflow: in fact, when modelled on a simulated PLA2R1 3D protein structure using cryo-EM volumes (courtesy of Dr. Paul Brenchley), a good correspondence between the PLA2R1 epitopes recognized by the coupled analyses and previously published studies^24,25,29^ could be demonstrated. Correlation of specific epitopes with proteinuria over time was also confirmed^23^. Again, non-PLA2R1 antigen targets of antibodies in MN, i.e*.* THSD7A^2^, NEP1^8^, NELL1^5^, SEMA3B^6^, PCDH7^9^ and HTRA1^7^, were not identified here, emphasising the non-alternative value of these techniques.

With this limitation in mind, we subsequently applied the peptide technology to tentatively discover novel autoantibodies in MN patient sera and uncovered their relevance. The main result was the identification of 21 proteins that differentiated MN from healthy people using multidimensional scaling (MDS) analysis and among these, anti-FMNL1 IgG4 emerged as circulating antibodies associated with MN clinical outcome.

FMNL1 is a protein belonging to the formin family, playing crucial roles in actin-dependent processes, including migration, vesicle trafficking, morphogenesis^30,31^ and leukocyte migration^32^. FMNL1 expressed in macrophages is probably a marker of a sub-population with functions yet to be characterized^33,34^. Findings obtained by peptide array were cross-validated using sandwich ELISA. Anti-FMNL1 antibodies, mostly of the IgG4 isotype as expected^35^, were present in sera of MN patients and were more abundant in PLA2R1 negative MN than in PLA2R1 positive MN. Immunohistochemistry on MN kidney biopsies showed that FMNL1 is expressed in glomerular circulating macrophages but not co-expressed with membranous IgG4 or PLA2R1 paralleling the serological assessment showing higher FMNL1 titer in PLA2R1 negative patients. To further strengthen the association of FMNL1 and macrophages, we blotted whole macrophage lysates obtained from healthy donors using sera from FMNL1 low and high titer MN patients. Results clearly indicated the presence of a band at the same level of recombinant FMNL1 only in FMNL1 high titer patients.

We also observed that anti-FMNL1 IgGs were not restricted uniquely to MN and high circulating levels were detected in patients with other glomerulonephritis such as idiopathic and genetic FSGS and IgAGN. Those findings are potentially suggestive of a broader role of those autoantibodies in other glomerular disease conditions.

Taken together, these data point toward the presence in MN patients with poor clinical outcome, of a novel class of circulating autoantibodies directed against cells other than podocytes. This is the first time such an observation is made in the context of MN. Previous studies in fact showed autoantibodies being directed against either the plasma membrane of podocytes (PLAR1 and others), thought to be involved in the initial sub-epithelial immune complex deposits and disease initiation, or intracellular proteins^3^ (such as superoxide dismutase) thought to be involved in disease progression^13,15,16^. Our study resulted in the identification of anti-FMNL1 macrophage-directed autoantibodies.

Whereas central role of M1^36^ and M2^37^ macrophages has been extensively studied in nephrotoxic serum (NTS) glomerulonephritis, a preclinical model of crescentic glomerulonephritis^38^, only limited scientific evidence exists supporting a potential role for macrophages in MN^39,40^. According to our observation of higher anti-FMNL1 levels in MN cases with no remission we could hypothesize that these anti-FMNL1 autoantibodies might be involved in some sort of secondary mechanisms related to normalisation of glomerular structure and function. Although very speculative, those antibodies might potentially exert their effect depleting a specific macrophage subpopulation involved in disease resolution. This hypothesis is supported by a recently published study that examined in detail the macrophage subtypes in MN and reported a differential role for the different macrophage subtypes, with M2c macrophages *negatively* correlating with MN outcome^40^. This hypothesis goes far beyond the scope of the present work and will require dedicated histological and clinical study analysing FMNL1 positive macrophages subtypes and correlating FMNL1 positivity with MN evolution (resolution or progression).

In conclusion, we have demonstrated that high-density peptide arrays coupled with an appropriate data analysis pipeline could be successfully deployed to identify novel MN autoantigens and that FMNL1, a protein expressed by macrophages, is a novel autoantigen in patients affected by MN and potentially involved in sustaining disease progression. The association of high anti-FMNL1 antibodies and absence of remission strengthens the concept that in MN, autoantibodies not directly involved in the disease onset might have a role in disease progression.

## Materials and methods

### Patients

The study comprised a total of 56 healthy donors and 211 patients (170 with MN, 23 with idiopathic focal segmental glomerulosclerosis, 6 with genetic nephrotic syndrome, 12 with IgA glomerulonehritis). Criteria for MN patients enrollment were: biopsy-proven diagnosis of membranous nephropathy associated with proteinuria > 0.3 g/d; normal complement profile; negative tests for anti-THSD7A, anti-nuclear antibodies, anti-double strand DNA, ANCA, cryoglobulins and the absence of viral markers (hepatitis B surface antigen and HIV); absence of clinical and biochemical signs of cancer; serum samples available at hospitalization and during follow-up (Italian Study Group on Membranous Nephropathy-EudraCT 2011-003942-41). All MN sera used in this study were tested for anti-PLA2R1 (Euroimmun, Lübeck, Germany). MN sera were collected at time of hospitalization (T_0_) and after 12 months (T_12_) or 24 months (T_24_). Serum of other categories were collected only at the time of diagnosis. Clinical characteristics of all categories patients are reported in Tables 1 and 2. Serum collection and analysis was approved by Comitato Etico Regione Liguria, an independent ethical committee, on October 14, 2014, study number: 408REG2014.

### Peptide array synthesis

Tiled peptide microarrays were synthesized with a Maskless Array Synthesizer (MAS) by light-directed solid-phase peptide synthesis using an amino-functionalized support coupled with a 6-aminohexanoic acid linker and amino acid derivatives carrying a photosensitive 2-(2-nitrophenyl) propyl oxycarbonyl (NPPOC) protection group. Amino acids (final concentration 20 mM) were pre-mixed for 10 min in N,N-Dimethylformamide (DMF, Sigma Aldrich) with *N*,*N*,*N*′,*N*′-Tetramethyl-O-(1H-benzotriazol-1-yl)uronium-hexafluorophosphate (HBTU, Protein Technologies, Inc.; final concentration 20 mM) as an activator, 6-Chloro-1-hydroxybenzotriazole (6-Cl-HOBt, Protein Technologies, Inc.; final concentration 20 mM) to suppress racemization, and *N*,*N*-Diisopropylethylamine (DIPEA, Sigma Aldrich; final concentration 31 mM) as base. Activated amino acids were then coupled to the array surface for 3 min. Following each coupling step, the microarray was washed with *N*-methyl-2-pyrrolidone (NMP, VWR International), and site-specific cleavage of the NPPOC protection group was accomplished by irradiation of an image created by a Digital Micro-Mirror Device, projecting 365 nm wavelength light. Coupling cycles were repeated to synthesize the full in silico-generated peptide library. Prior to sample binding, final removal of side-chain protecting groups was performed in 95% trifluoroacetic acid (TFA, Sigma Aldrich), 0.5% Triispropylsilane (TIPS, TCI Chemicals) for 30 min. Arrays were incubated in methanol for 30 s and rinsed 4 times with reagent-grade water. Arrays were washed for 1 min in TBS and 0.05% Tween-20 (TBS-T), 2 times for 1 min in TBS, and exposed to a final wash for 30 s in reagent-grade water. The whole proteome peptide array contained the entire genome synthesized as a total of 7,499,126 peptides with a tiling of 3 amino acids. Specifically, for PLA2R1 protein, the array was synthesized with a higher resolution with single amino acid tiling. Data of whole proteome peptide discovery array is friendly available at https://github.com/cavallilab/fullproteome.

### Sample binding and detection

Samples were diluted 1:100 in TBS-T and 1% alkali-soluble casein and bound to arrays overnight at 4 °C. After sample binding, the arrays were washed 3 times in TBS-T, 10 min per wash. Primary sample binding was detected via Alexa Fluor® 647-conjugated goat anti-human IgG secondary antibody (Jackson ImmunoResearch). The secondary antibody was diluted 1:10,000 (final concentration 0.1 ng/µl) in TBS-T and 1% alkali-soluble casein. Arrays were incubated with secondary antibody for 3 h at room temperature, then washed 3 times in wash TBS-T, washed for 30 s in reagent-grade water, and then dried by spinning in a microcentrifuge equipped with an array holder. Fluorescent signal of the secondary antibody was detected by scanning at 635 nm at 2 µm resolution and 15% gain, using an MS200 microarray scanner (Roche NimbleGen). Scanned array images were analysed with proprietary Roche software to extract fluorescence intensity values for each peptide.

### Bioinformatics analysis

The sliding window probabilistic identification of epitopes (S-PIE) is based on the observation that for true epitopes a signal above a given threshold should be detectable for all consecutive peptides containing the recognized amino acids sequence (Fig. 1A). Therefore, for a cut-off fluorescence intensity I_0_, we first search for regions with at least two adjacent peptides with an intensity I > I_0_. Then, we compute the ratio, R, between the probability of having N consecutive intensities larger that the cut-off, P(I_a_…I_n_), and the probability of having the intensity larger than the cut-off N times, P(I_a_)…P(I_n_), where probabilities are estimated using all measured intensities. The value R is then assigned to all peptides of the window under consideration. The analysis is then repeated for increasing values of I_0_ and, for each peptide the latest value of R is kept. Finally, all peptides with a value of R > 10^6^ were selected as potential hits (Fig. 1B).Peptides with a sequence that contained a significant number of repeated amino acids were eliminated.

In the weight gene co-expression network analysis (WGCNA)^41^ immuno-intensity data were filtered removing all peptides with the mean intensity of the healthy was greater than the MN patients. Tiled peptide microarray data were analysed by unsupervised hierarchical clustering using multidimensional scaling (MDS) with k-means and Spearman’s correlation to identify outliers and dissimilarity between samples. The normalized autoantibodies titer profiles of the peptides were then used to construct the co-expression network using the WGCNA.A weighted adjacency matrix was constructed using the power function. After choosing the appropriate β parameter of power (with the value of independence scale set to 0.9) the adjacency matrix was transformed into a topological overlap matrix (TOM), which measures the network connectivity of all the peptides. To classify peptides with similar autoantibodies titer profiles into peptide modules, hierarchical clustering analysis was conducted according to the TOM dissimilarity with a minimum of 30 peptides per module. To identify the relationship between each module and clinical trait, we used module eigengenes (MEs) and calculated the correlation between MEs and each clinical trait and their statistical significance corrected for multiple interactions. A heat map was then used to visualize the degree of each relationship (Fig. 1C). The MEs/peptides with a significant relationship with the prediction of proteinuria at T_12_and T_24_were selected.

### B-cell epitope prediction analysis

The B-cell epitope prediction for PLA2R1and FMNL1 were performed using BepiPred-2.0^42^ using the sequence from the UNIPROT database Q13018 and O95466, respectively. The BepiPred-2.0 server predicts B-cell epitopes from a protein sequence, using a Random Forest algorithm trained on epitopes and non-epitope amino acids determined from crystal structures. The residues with scores above the threshold 0.55 were predicted to be part of an epitope. All peptides reported in Supplemental Fig. 1 (PLA2R1) and in Supplemental Fig. 2 (FMNL1) are above the threshold (> 0.55).

### ELISA assay for anti-FMNL1

To test circulating autoantibodies levels in serum, we designed an ELISA assay using recombinant whole FMNL1 protein. Briefly, 20 ng/well of FMNL1 whole recombinant protein (Lifespan Biosciences, Seattle, Washington, USA) were coated to 96-well maxisorp nunc-immuno plates (ThermoFisher Scientific, MA, USA) overnight at 4 °C. After blocking in 3% w/v BSA in PBS, 100 μl 1:100 diluted samples per well were incubated overnight at 4 °C. Samples were removed, and wells were washed three times with PBS and 0.05% v/v of tween-20 (PBS-T). Then, plate was incubated 4 h with the anti-human IgG4 HRP conjugated (1:2000 diluted in 1% w/v BSA in PBS-T). At the end of time the samples were wash again three times with PBS-T. Finally, the peroxidase substrate (TMB, Bio-Rad, Hercules, CA, USA) was added, and the absorbance at 450 nm wavelength was measured using xMark microplate Absorbance Spectrophotometer (Bio-Rad). The results were expressed as Relative Unit for ml (RU/ml). The Kruskal–Wallis test was used to assess differences in the auto-antibodies levels among the different comparison considered in this study. The medians and interquartile range (IQr) of each group were reported. A value of *P* < 0.05 was considered to be statistically significant. Received operating characteristic (ROC) curves were generated to assess the diagnostic efficiency of each assay.

### Western blotting analysis

Characterization of macrophage antigens recognized by serum of MN patients were performed by western blot. Recombinant FMNL1 (0.5 µg, Lifespan Biosciences, Seattle, Washington, USA) or whole lysate of macrophage (20 µg for each lane) were solubilized in 2% w/v SDS, 10% glycerol, 50 mM DTE, and 62.5 mM Tris–HCl pH 6.8 and separated by sodium dodecyl sulfate polyacrylamide gel electrophoresis (SDS-PAGE) and then transferred to a nitrocellulose membrane. Membrane was stained with a solution of 0.25%w/v Ponceau, 1%v/v methanol and 1%v/v acetic acid and cut perpendicular to the electrophoresis migration front to obtain a full length strips of whole samples and to allow the individually labelled and detected with different antibodies or sera. Ponceau staining was removed with H_2_O and the membrane strips were blocked with 3% w/v bovine serum albumin (BSA) in PBS. After rinsing in PBS containing 0.05% v/v Tween-20 (PBS-T), membrane strips were detected with human anti-actin (Sigma Aldrich) or human anti-FMNL1 or healthy donors or MN patients serum diluted in 3% w/v BSA in PBS-T. After rinsing in PBS-T, the membrane strips were incubated with HRP-conjugated secondary antibodies (diluted 1:10,000 in 1% w/v BSA in PBS-T) and developed at the same time. Chemiluminescence signal was acquired and quantified using respectively the ChemiDoc and Quantity One software (Bio-Rad, Hercules, CA, USA).

### Human renal tissues

Human renal tissue, fixed in formaldehyde and embedded in paraffin, was selected from the files of the Service of Pathology, University Hospital Geneva: 5 control normal renal tissues were obtained from patients with nephrectomy performed for neoplasia and 23 renal biopsies from patients with membranous nephropathy (8 PLA2R1 positive MN, 1 idiopathic PLA2R1 negative MN, 14 secondary MN whose 12 patients with lupus nephritis and 2 patients with neoplasia). For all biopsy specimens, standard analysis using light microscopy, immunofluorescence (with anti-immunoglobulin IgA, IgG, IgM, anti-light chains kappa and lambda, and anti-complement C1q, C3, C4c, and C5b-9 antibodies), and electron microscopy was performed. Each patient gave informed consent before enrolment. The institutional ethical committee board approved the clinical protocol (CEREH Number 03-081). The research was performed according to the Helsinki’s declaration principles.

### Immunohistochemistry on human tissues

Immunohistochemistry was performed as follows: after antigen heat retrieval, 3 μm sections of the formaldehyde-fixed, paraffin-embedded biopsy specimens were incubated with rabbit polyclonal anti-human FMNL1 antibody (Abcam, Cambridge, UK) at a 1/200 dilution 1 h at room temperature followed by an anti-rabbit antibody for 30 min (room temperature) and then liquid diaminobenzidine substrate–chromogen system (DakoCytomation, Glostrup, Denmark).

For glomerular localization experiments in normal kidney and MN, serial paraffin sections were submitted to the appropriate antigen retrieval and stained using each of these different primary antibodies: anti-FMNL1 (Abcam) at 1/200 dilution, anti-PLA2R1 (Abcam) at 1/1000 dilution, anti-IgG4 (Merck, California, USA) at 1/50 dilution, anti-CD3 (Roche Diagnostics Corporation, Indiana, USA) at 1/100 dilution, anti-CD79a (DakoCytomation) at 1/50 dilution and anti-CD68 (DakoCytomation) at 1/500 dilution.

For glomerular co-localization experiments in MN, double immunostaining with FMNL1 and CD68 antibodies was performed on paraffin-embedded sections (3 μm thick) as previously reported^43^. Briefly, 3 μm sections of paraffin-embedded kidneys were submitted to antigen heat retrieval and incubated with anti-FMNL1 antibody (Abcam) at 1/200 dilution 1 h at room temperature followed by an anti-rabbit antibody for 30 min (room temperature) and then liquid diaminobenzidine substrate–chromogen system (DakoCytomation). Sections were then incubated with anti-CD68 (DakoCytomation) at 1/500 dilution for 1 h at room temperature followed by the appropriate second antibody for 30 min and then by phosphatase alkaline-fast red enzyme system (DakoCytomation). Stained sections were examined with a Zeiss microscope.

### Statistical methods

A combination of T-test, Partial Least Squire Discriminant Analysis (PLS-DA) and Support Vector Machine learning (SVM) was utilized to maximize the difference in Fluorescence. With T-test, *P*-values were adjusted using the method of Benjamini–Hochberg for multiple interaction. Peptides were considered statistically significant when displaying an adjusted *P*-value ≤ 0.05 and a fold time change ≥ 2. Volcano plot visualized the statistical differences obtained with T-test analysis. The cutoff of significance was indicate with a black lines using the function y = c/(x − x0).

SVM utilized the ANOVA method to optimize the peptides selection and the four-fold cross-validation approach to estimate the prediction and classification accuracy. The whole dataset was randomly divided into two parts, one for learning (65% of data) and the other one (35%) to test the accuracy of prediction. The training was repeated until all possible combination of the subjects in the two groups are done (Origin Lab software). This analysis identified a peptides rank score that ranged between 0 (maximal power discrimination) and 18,757 (minimal power discrimination).

PLS-DA identified for each peptide a variables of importance called VIP score that maximized the discrimination power among the comparative procedures (MN vs healthy). The profiles of the highlighted peptides deriving from the combination of the three analysis above (T-test, SVM, PLS-DA) were visualized using the heatmap diagram. In the heatmap each row represents a peptide and each column corresponds to a clinical group. Normalized Z-scores of peptides intensity are depicted by a pseudocolor scale (i.e*.* blue, white and red). The tree dendrogram displays the results of unsupervised hierarchical clustering analysis, placing similar clinical group/peptide intensity values next to each other.

To compare serum levels of FMNL1 obtained with ELISA, the Mann–Whitney (two samples) or Kruskal–Wallis (more than two samples) test were used. Results were expressed as medians and interquartile range (IQr). For Mann-Withney a value of *P* < 0.05 was considered statistically significant, while for Kruskal–Wallis a value of *P* < 0.05 after Dunns correction was considered statistically significant. Receiver operating characteristic (ROC) curves were generated to assess the diagnostic efficiency of each comparison. Youden's index and Likelihood ratio^44^ were used, respectively to identify the cutoff and the diagnostic performance of the tests. Statistical analyses were performed using the last version of software package R available at the time of the experiments and OriginLab software.

## Supplementary Information

Supplementary Information 1.

Supplementary Information 2.

## Data Availability

All data generated or analyzed during this study are included in this article with the exception of whole proteome peptide discovery array that is friendly available at https://github.com/cavallilab/fullproteome.
