# Supplementary material for: Discovery of anti-Formin-like 1 protein (FMNL1) antibodies in membranous nephropathy and other glomerular diseases
Source: Sci Rep. 2022 Aug 11;12:13659. doi: 10.1038/s41598-022-17696-w (PMC9372176; doi:10.1038/s41598-022-17696-w)
Supplement: Supplementary file 1 — Supplementary Information 1. [file 41598_2022_17696_MOESM1_ESM.docx]

**Supplemental Materials**

**Discovery of anti-Formin-like 1 Protein (FMNL1) antibodies in proteinuric nephropathies.**

Maurizio Bruschi^1,#^, Andrea Cavalli^2,3,#^, Solange Moll^4^, Giovanni Candiano^1^, Leonardo Scapozza^7^, Jigar J. Patel^5^, John C. Tan^5^, Ken C. Lo^5^, Andrea Angeletti^6^, Gian Marco Ghiggeri^6,§*^ and Marco Prunotto^7,§^

^1^Laboratory of Molecular Nephrology and Unit of Nephrology, Dialysis and Transplantation, IRCCS IstitutoGianninaGaslini, Genoa, Italy

^2^Institute for Research in Biomedicine, Università della Svizzera Italiana, Bellinzona, Switzerland

^3^Swiss Institute of Bioinformatics, Lausanne, Switzerland

^4^Department of Pathology, University Hospital of Geneva, Switzerland

^5^Nimble Therapeutics, Madison, WI, USA

^6^Division of Nephrology, Dialysis and Transplantation, IRCCS IstitutoGianninaGaslini, Genoa, Italy

^7^Institute of Pharmaceutical Sciences of Western Switzerland, University of Geneva, Geneva, Switzerland

^#^equally contributing authors

^§^equally contributing last authors

**SupplementalFigures**


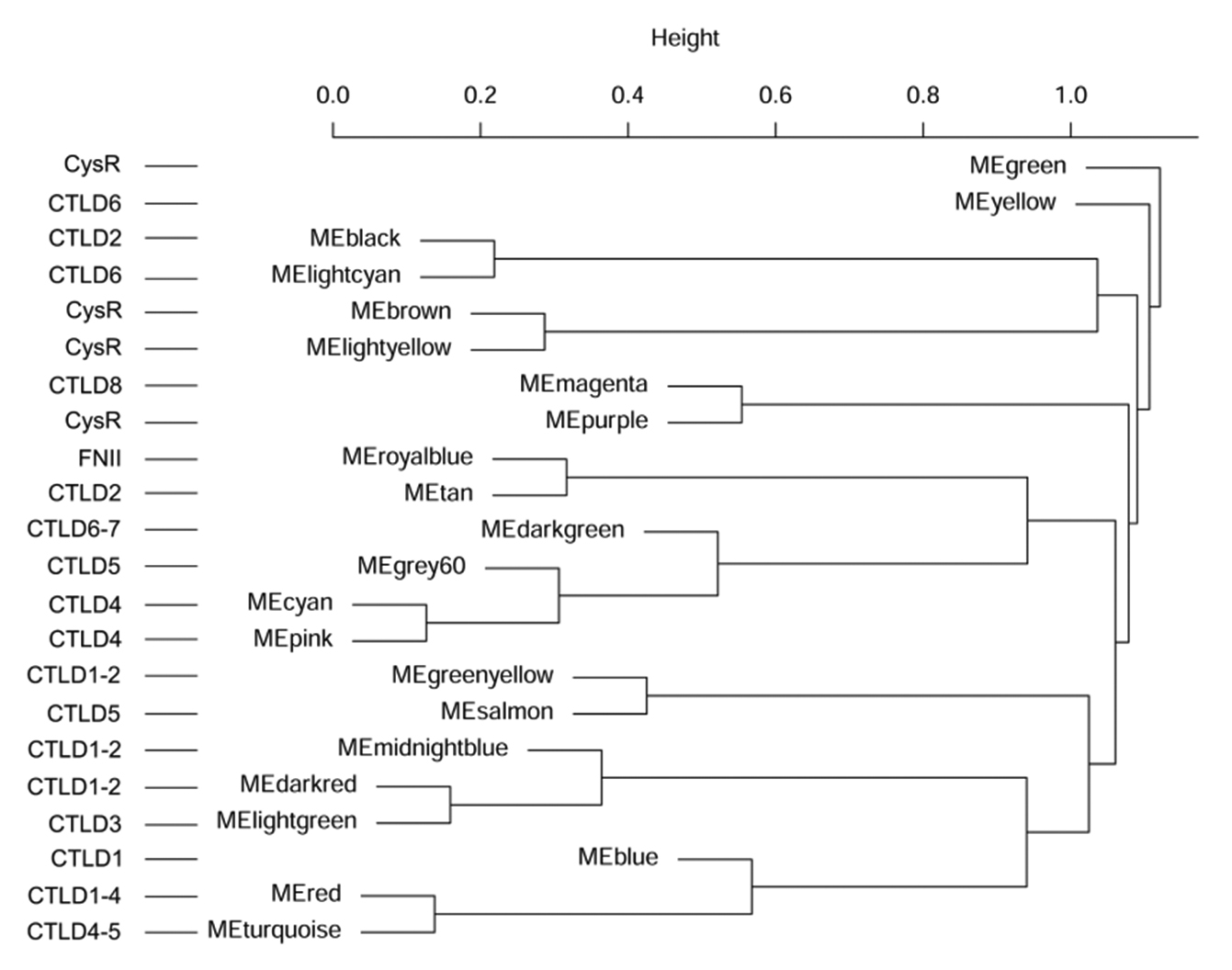


**Supplemental Figure 1. Fisher enrichment analysis of PLA2R1 WCGNA-identified modules**. The diagram shows Fisher's enrichment analysis of PLA2R1 epitopes in the identified modules of WGCNA. Modules related to proteinuria outcome at 12 and 24 months (blue, red and turquoise) are enriched in the first 5 CTLD domains of PLA2R1 proteins; green and yellow modules that correspond to CysR did not correlate with the same clinical data (proteinuria T0, T12, T24)

**
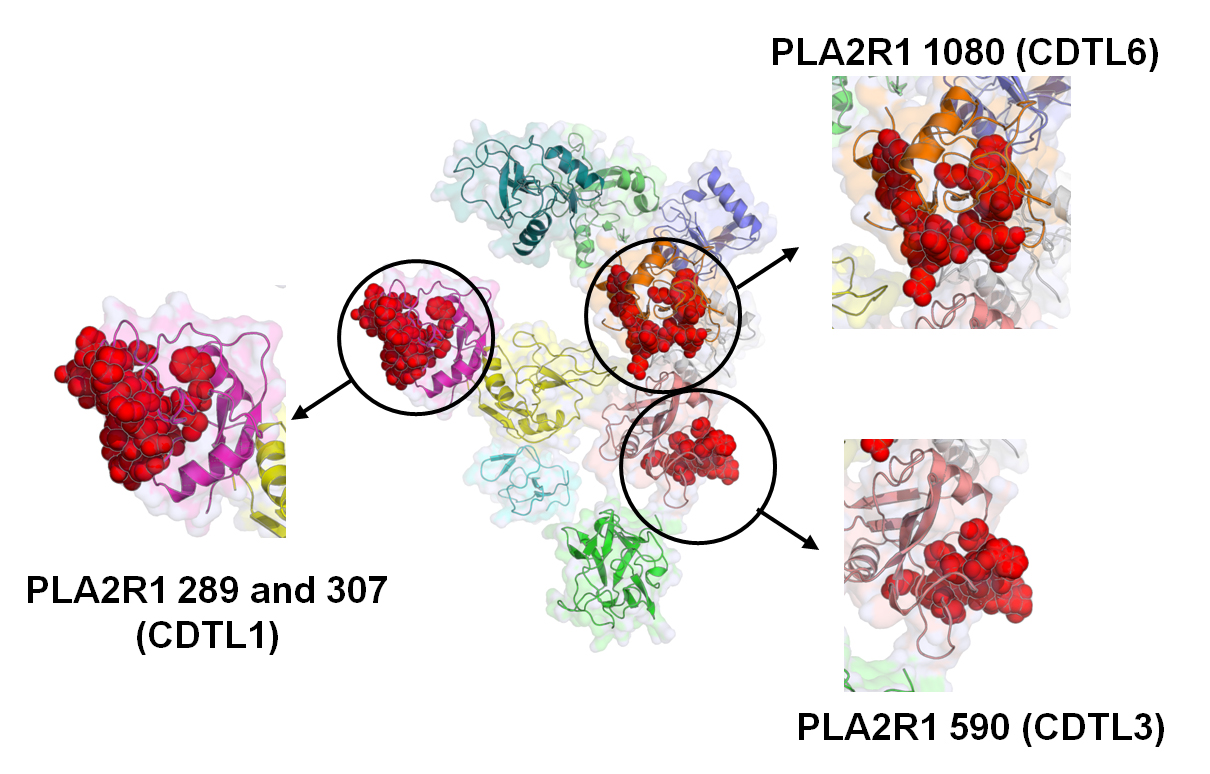
**

**Supplemental Figure 2**. **PLA2R epitopes** identified by the combine use of WGCNA and S-PIE methods mapped on a cryo-EM model of PLA2R. B-cell epitopes are coloured in red.

**
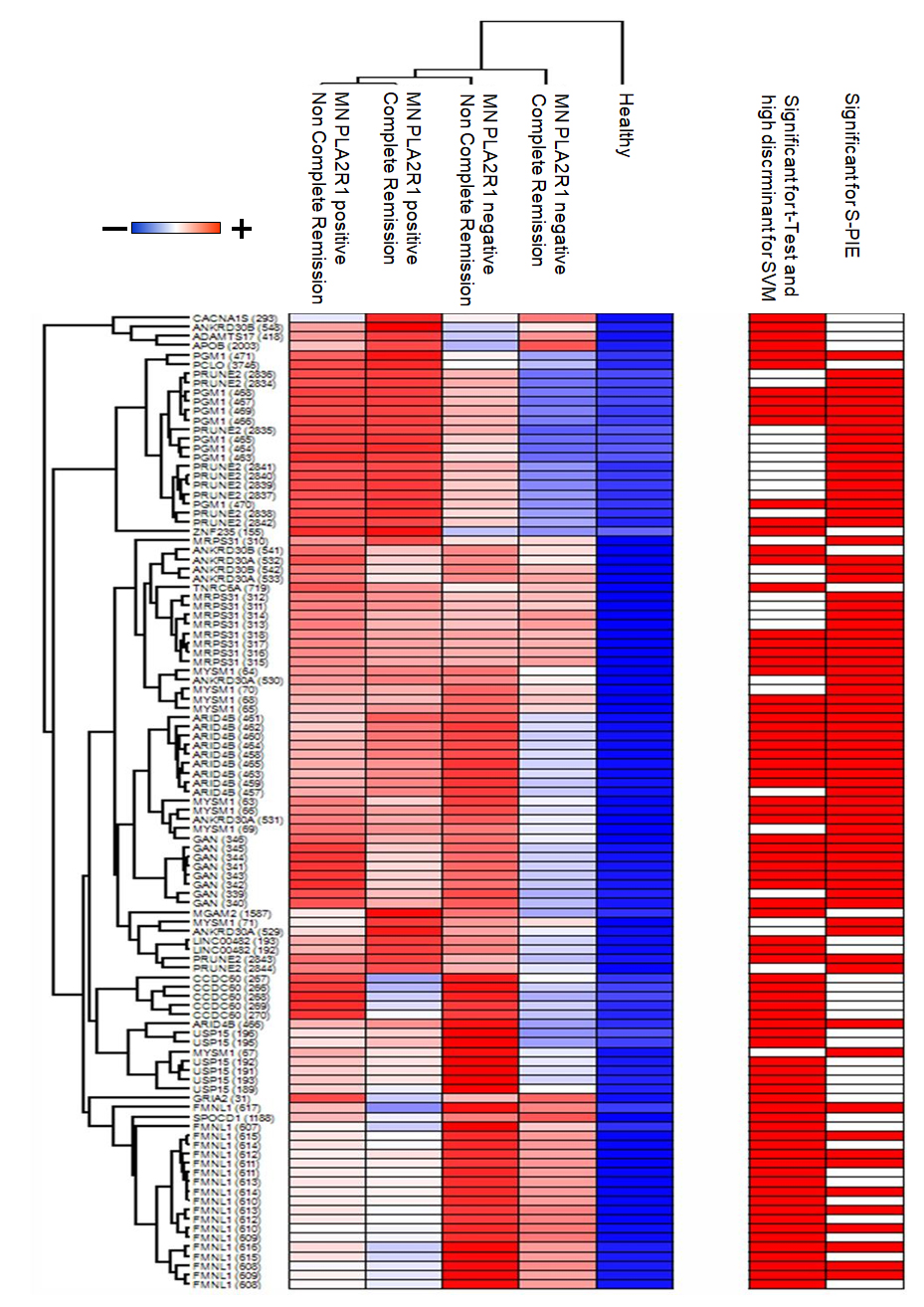
**

**Supplemental Figure 3. Heatmap of 105 peptides highlighted through the combined use of univariate statistical analysis, support vector machine learning and S-PIE analysis in all the comparison**. In the heatmap, each row represents a peptide and each column corresponds to a clinical condition. Normalized Z-scores of peptide abundance are depicted by a pseudocolour scale with red indicating positive expression, white equal expression and blue negative expression compared to each peptide value, whereas the dendrogram (on the top and left) displays the outcome of unsupervised hierarchical clustering analysis, placing similar sample/peptide profile values near each other. Visual inspection of the dendrogram and heatmap demonstrates the ability of these peptides to clear discriminate between the different conditions.

**
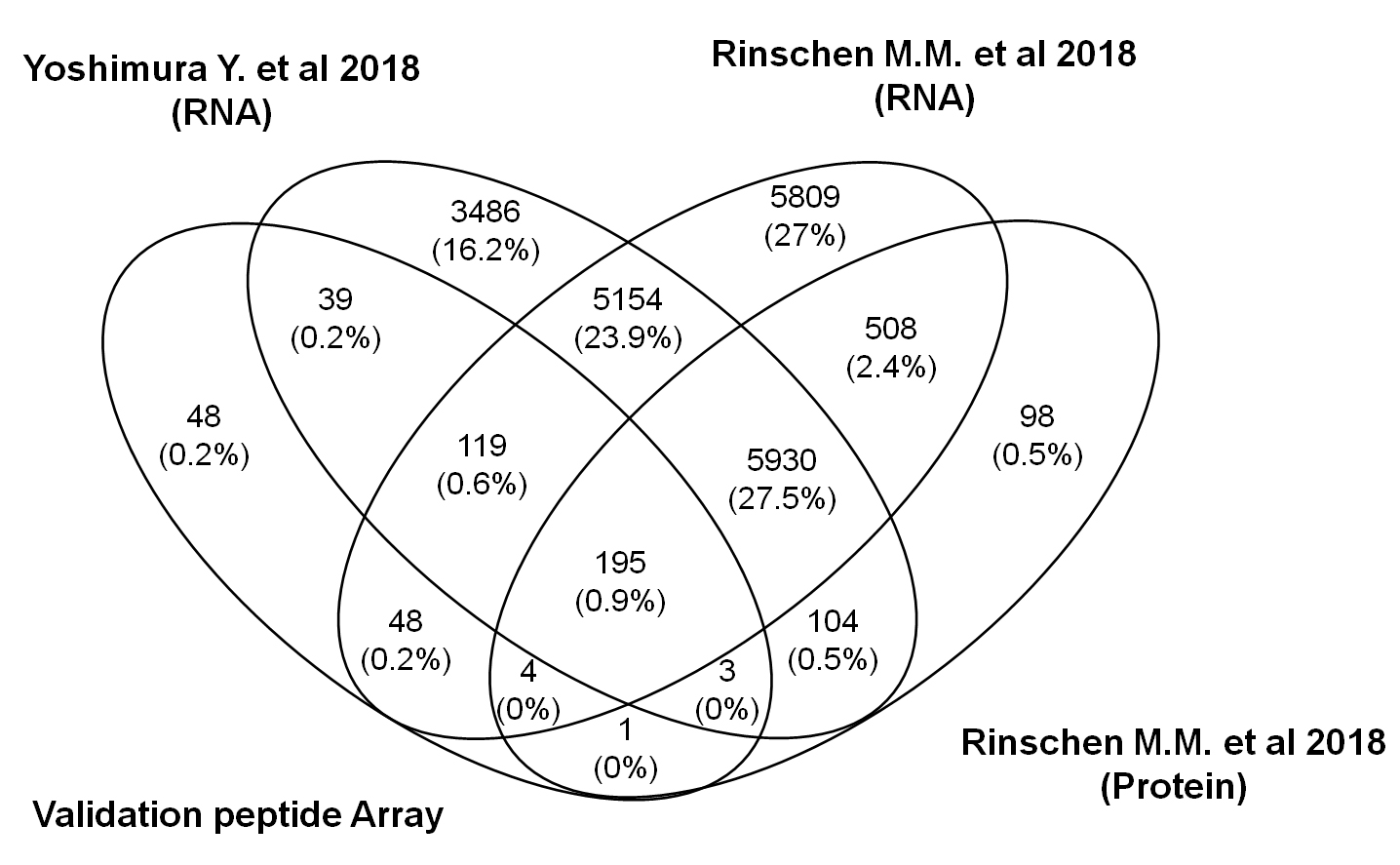
**

**Supplemental Figure 4. Venn diagram of validation peptides array.** Venn diagram shows the common and exclusive corresponding proteins of the validation peptides array and proteins identified in sorted human (26) and mouse podocytes(45). The vast majority (81%) of proteins selected for the validation array overlapped in human and/or mouse podocyte proteins.

**
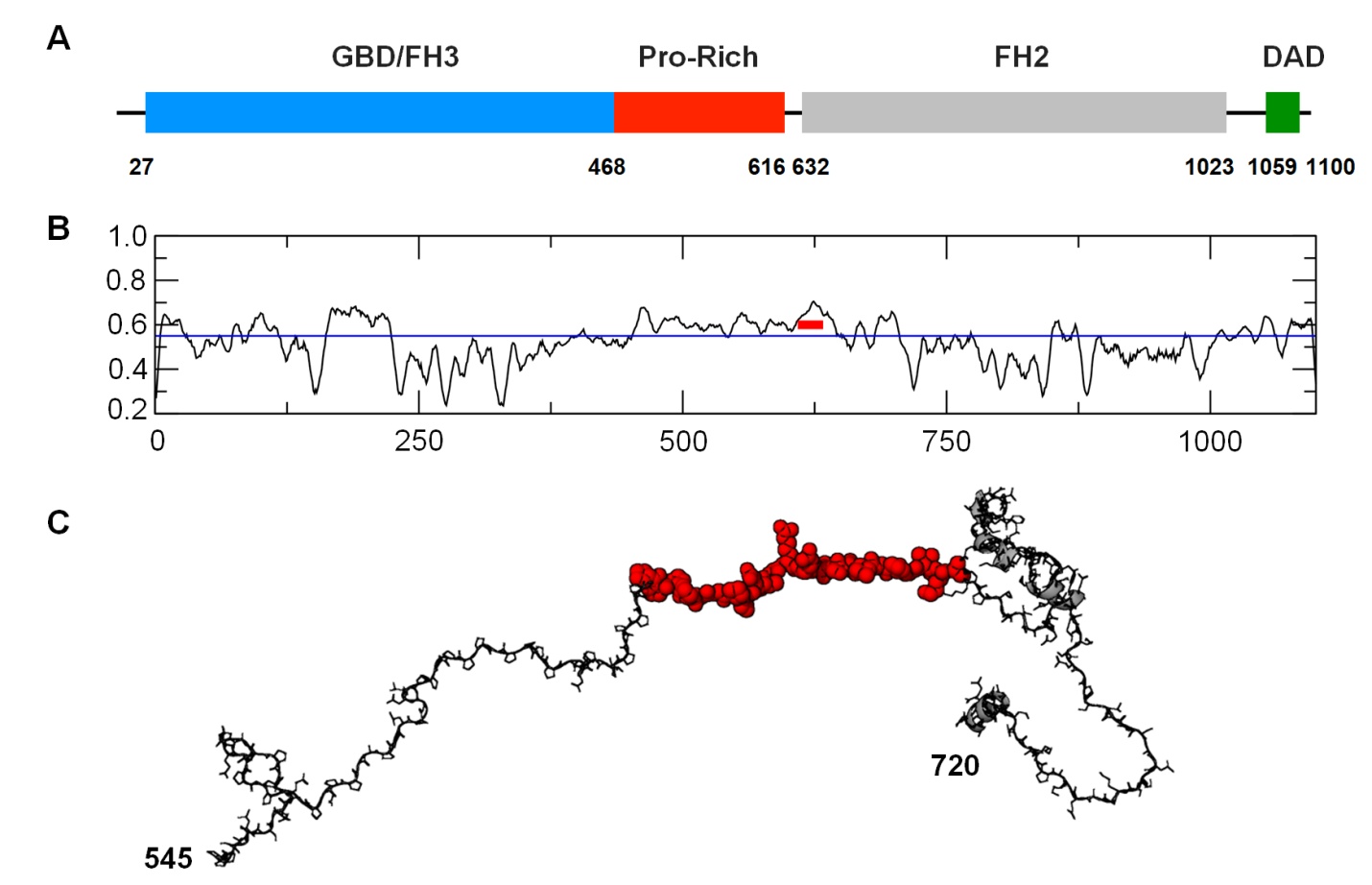
**

**Supplemental Figure 5. FMNL1 epitope** (A) Schematic representation of the domains of FMNL1. (B) BepiPred 2.0 B-cell epitope propensity. The peptide 609-639 if highlighted in red. (C) Structural model of the segment 545-720 of FMNl1 obtained with HHPED. Region 609-639 id represented with red van der Waals spheres.


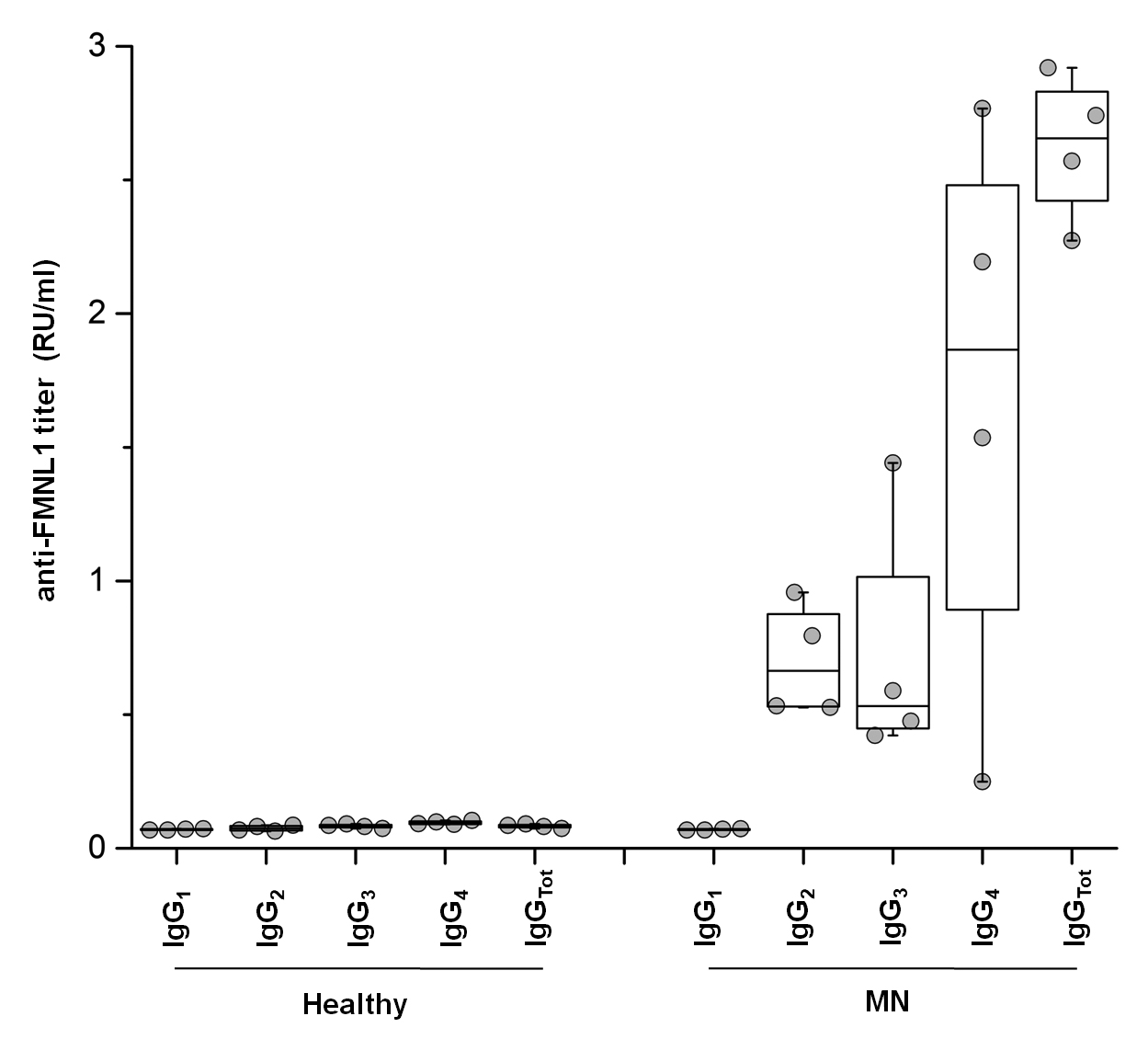


**Supplemental Figure 6. Characterization of serum antibodies isotypes for FMNL1.** Isotypes titre were characterized by ELISA assay. Four randomized healthy donors and MN patients positive for anti-FMNL1 ELISA assay were characterized for their isotype titre. MN isotypes titre show a high positivity of igG4 and weak positivity for IgG2 and 3. Whereas, healthy donors show no signal for each isotype. IgG tot were used as positive controls.

**
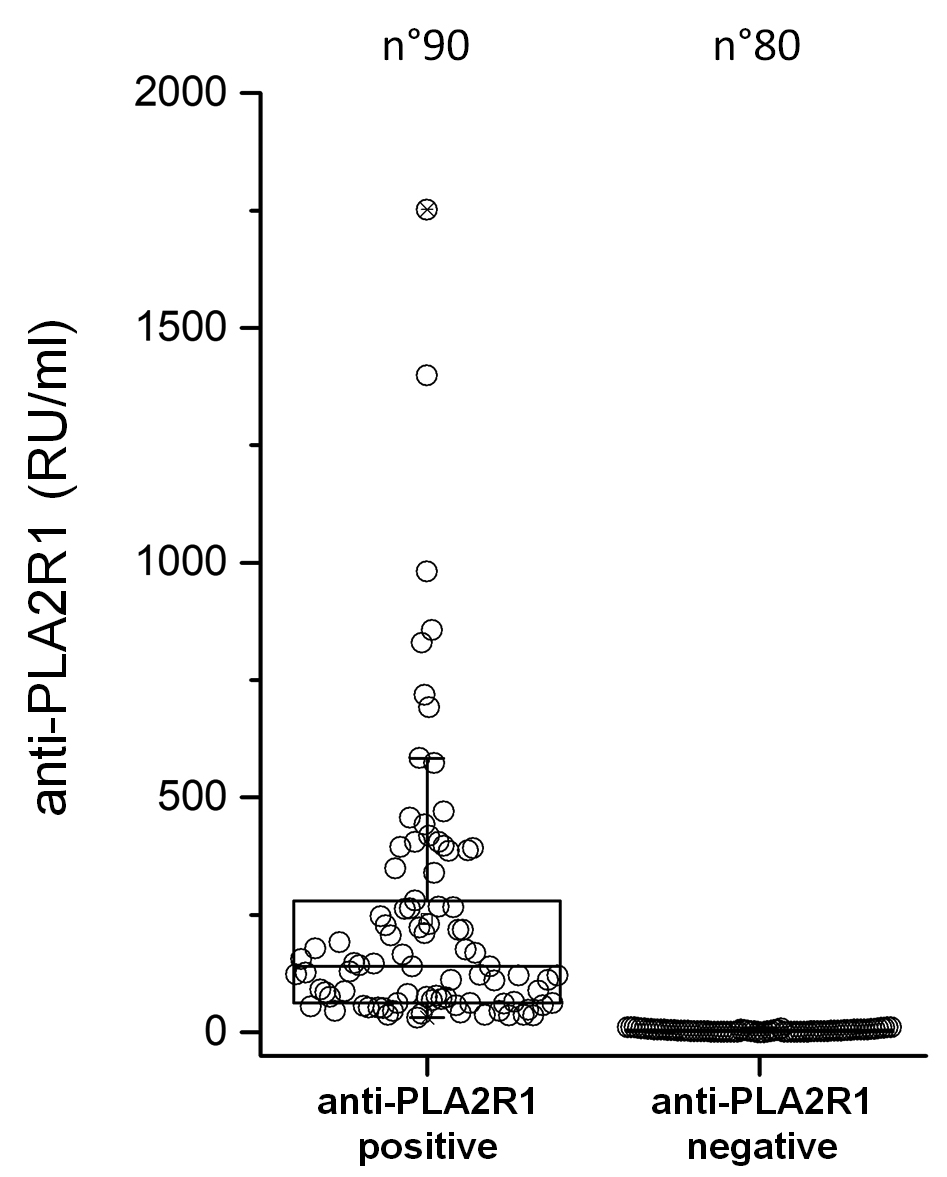
**

**Supplemental Figure 7. Serum anti-PLA2R1 titer in MN patients.** Detection of serum levels of anti-PLA2R1 antibodies was measured with a commercial ELISA kit (Euroimmune, Lübeck, Germany). The positivity cut-off was 20 RU/ml as given by manufacturer instruction.

**
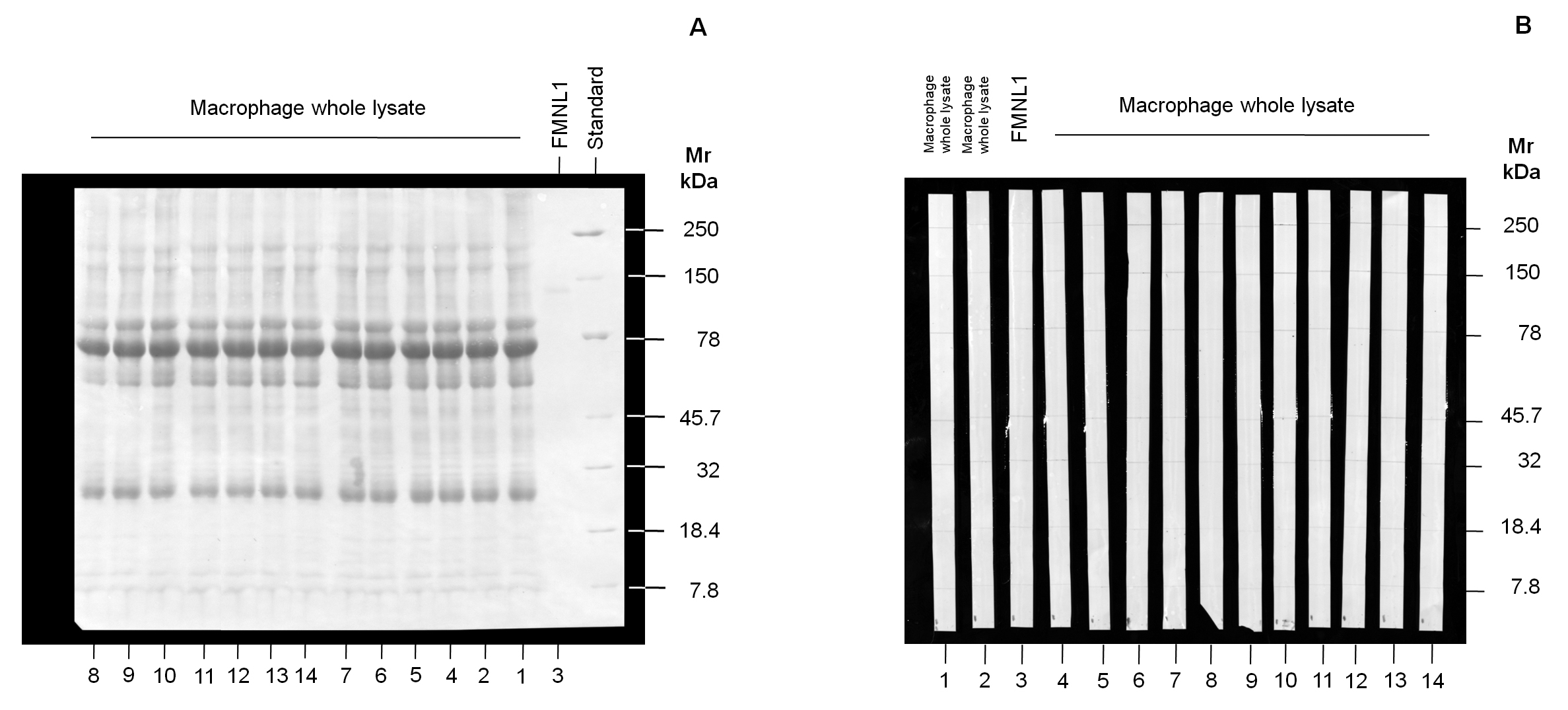
**

**Supplemental Figure 8. Ponceau staining and blank of western blot of full length DSD-PAGE of macrophage whole lysate.** (A) Image of Ponceau staining of western blot membrane showing total proteins loading from macrophage whole lysate before its cut, perpendicular to the electrophoresis migration front, to allow the individually labelled ad detection of each lane with different antibodies or human sera. (B) Blank image of western blot reported in Figure 5. The number under the Ponceau and blank corresponds to the same lane reported in Figure 5.

**Supplemental Tables**

**Supplemental Table 1. Validation peptide array data**. Data report the fluoresce intensity (FI) and the results of S-PIE, T-test and receive operating characteristic (ROC) curve analysis. For each peptide is reported Log2 FI, area under the curve (AUC), fold change and the corresponding -Log10 P-value of the different comparison. The symbol "+" indicate the peptides selected by the different types of analysis utilised. (attached as Supplemental Tables 1-2 xlsx file)

**Supplemental Table 2. PLA2R1 peptide array data.**Data report the fluoresce intensity and the results of S-PIE, Support Vector Machine (SVM) and Weight Gene Co-expression Network Analysis (WGCNA). For each peptide is reported module color, epitope, Spearman’s correlation coefficient and its -Log10 P-value. The symbol "+" indicate the peptides selected by the different types of analysis utilised. (attached as Supplemental Tables 1-2 xlsx file)

**Supplemental Table 3. ROC curve analysis**. Table of Receiver Operating Characteristic curve analysis of 21 proteins selected from validation array to maximize the discrimination between MN patients positive or negative for anti-PLA2R1 and healthy donors.

| **Proteins** | **n° of peptides** | **AUC Healthy vs MN** | **P-value Healthy**  **vs MN** | **AUC Healthy**  **vs MN PLA2R1**  **negative** | **P-value Healthy vs MN PLA2R1**  **negative** | **AUC Healthy vs MN PLA2R1**  **positive** | **P-value Healthy vs MN PLA2R1**  **positive** |
| --- | --- | --- | --- | --- | --- | --- | --- |
| **ADAMTS17** | 1 | 0.71 | 4.69 | 0.68 | 2.84 | 0.73 | 5 |
| **ANKRD30A** | 5 | 0.73 | 4.34 | 0.73 | 2.55 | 0.73 | 4.08 |
| **ANKRD30B** | 3 | 0.71 | 3.82 | 0.72 | 2.51 | 0.72 | 3.71 |
| **APOB** | 1 | 0.7 | 3.71 | 0.65 | 1.95 | 0.73 | 4.09 |
| **ARID4B** | 10 | 0.74 | 4.95 | 0.76 | 3.66 | 0.73 | 3.98 |
| **CACNA1S** | 1 | 0.71 | 3.7 | 0.74 | 2.99 | 0.69 | 3.2 |
| **CCDC60** | 5 | 0.74 | 4.91 | 0.72 | 3.04 | 0.75 | 4.69 |
| **FMNL1** | 19 | 0.74 | 4.61 | 0.81 | 4.41 | 0.71 | 3.11 |
| **GAN** | 8 | 0.75 | 6.26 | 0.74 | 3.07 | 0.76 | 5.6 |
| **GRIA2** | 1 | 0.72 | 4.03 | 0.71 | 2.39 | 0.73 | 3.83 |
| **LINC00482** | 2 | 0.71 | 3.91 | 0.7 | 2.84 | 0.72 | 3.6 |
| **MGAM2** | 1 | 0.71 | 3.61 | 0.73 | 2.55 | 0.7 | 3.13 |
| **MRPS31** | 9 | 0.71 | 3.39 | 0.69 | 2.4 | 0.72 | 3.73 |
| **MYSM1** | 9 | 0.74 | 5.09 | 0.76 | 3.79 | 0.74 | 4.29 |
| **PCLO** | 1 | 0.72 | 4.22 | 0.65 | 1.6 | 0.75 | 4.56 |
| **PGM1** | 9 | 0.72 | 4.8 | 0.65 | 1.65 | 0.75 | 5.42 |
| **PRUNE2** | 11 | 0.71 | 4.75 | 0.67 | 2.1 | 0.73 | 4.71 |
| **SPOCD1** | 1 | 0.7 | 3.41 | 0.73 | 2.8 | 0.69 | 2.75 |
| **TNRC6A** | 1 | 0.71 | 3.75 | 0.66 | 1.79 | 0.74 | 4.29 |
| **USP15** | 6 | 0.75 | 5.1 | 0.79 | 4.32 | 0.73 | 3.89 |
| **ZNF235** | 1 | 0.68 | 3.41 | 0.57 | 0.72 | 0.73 | 4.67 |

**Supplemental Table 4.** Clinic characteristics data of analysed patient biopsies.

| **Patient ids** | **Age**  **(year)** | **Gender** | **Screat**  **(mmol/l)** | **Proteinuria**  **(g/24h)** | **Heamaturia** | **anti-PLA2R1** | **Etiology** |
| --- | --- | --- | --- | --- | --- | --- | --- |
| 1 | 47 | M | 108 | 5.3 | positive | positive | primary |
| 2 | 76 | M | 139 | 7 | negative | positive | primary |
| 3 | 68 | M | 100 | 3 | negative | positive | primary |
| 4 | 26 | M | 86 | 2.8 | positive | positive | primary |
| 5 | 64 | M | 80 | 2.5 | negative | positive | primary |
| 6 | 41 | M | 71 | 8 | positive | positive | primary |
| 7 | 45 | F | 58 | 11 | positive | positive | primary |
| 8 | 57 | F | 58 | 8.5 | positive | positive | primary |
| 9 | 54 | M | 80 | 5 | negative | negative | primary |
| 10 | 70 | M | 560 | 10 | negative | negative | neoplasia |
| 11 | 61 | M | 98 | 3 | negative | negative | neoplasia |
| 12 | 31 | F | 61 | 3 | negative | negative | SLE class V |
| 13 | 18 | F | 45 | 5 | negative | negative | SLE class V |
| 14 | 39 | F | 61 | 5.5 | positive | negative | SLE class V |
| 15 | 27 | F | 88 | 6.3 | negative | negative | SLE class V |
| 16 | 32 | F | 115 | 5.4 | positive | negative | SLE class V |
| 17 | 45 | F | 63 | 3 | negative | negative | SLE class III+V |
| 18 | 43 | F | 60 | 2.5 | positive | negative | SLE class III+V |
| 19 | 22 | F | 100 | 2 | positive | negative | SLE class III+ V |
| 20 | 24 | F | 67 | 6.3 | positive | negative | SLE class III+V |
| 21 | 35 | F | 54 | 2.7 | positive | negative | SLE class III+V |
| 22 | 44 | M | 62 | 4.8 | positive | negative | SLE class III+V |
| 23 | 36 | F | 191 | 6.7 | positive | negative | SLE class IV+V |
